# The gut microbiota in conventional and serrated precursors of colorectal cancer

**DOI:** 10.1186/s40168-016-0218-6

**Published:** 2016-12-30

**Authors:** Brandilyn A. Peters, Christine Dominianni, Jean A. Shapiro, Timothy R. Church, Jing Wu, George Miller, Elizabeth Yuen, Hal Freiman, Ian Lustbader, James Salik, Charles Friedlander, Richard B. Hayes, Jiyoung Ahn

**Affiliations:** 1 Department of Population Health, New York University School of Medicine, New York, NY USA; 2 Division of Cancer Prevention and Control, Centers for Disease Control and Prevention, Atlanta, GA USA; 3 Division of Environmental Health Sciences, School of Public Health, University of Minnesota, Minneapolis, MN USA; 4 Department of Surgery, New York University School of Medicine, New York, NY USA; 5 Department of Cell Biology, New York University School of Medicine, New York, NY USA; 6 NYU Perlmutter Cancer Center, New York University School of Medicine, New York, NY USA; 7 Kips Bay Endoscopy Center, New York, NY USA

**Keywords:** Microbiome, Microbiota, Adenoma, Polyp, Colorectal, Cancer, Serrated

## Abstract

**Background:**

Colorectal cancer is a heterogeneous disease arising from at least two precursors—the conventional adenoma (CA) and the serrated polyp. We and others have previously shown a relationship between the human gut microbiota and colorectal cancer; however, its relationship to the different early precursors of colorectal cancer is understudied. We tested, for the first time, the relationship of the gut microbiota to specific colorectal polyp types.

**Results:**

Gut microbiota were assessed in 540 colonoscopy-screened adults by 16S rRNA gene sequencing of stool samples. Participants were categorized as CA cases (*n* = 144), serrated polyp cases (*n* = 73), or polyp-free controls (*n* = 323). CA cases were further classified as proximal (*n* = 87) or distal (*n* = 55) and as non-advanced (*n* = 121) or advanced (*n* = 22). Serrated polyp cases were further classified as hyperplastic polyp (HP; *n* = 40) or sessile serrated adenoma (SSA; *n* = 33). We compared gut microbiota diversity, overall composition, and normalized taxon abundance among these groups.

CA cases had lower species richness in stool than controls (*p* = 0.03); in particular, this association was strongest for advanced CA cases (*p* = 0.004). In relation to overall microbiota composition, only distal or advanced CA cases differed significantly from controls (*p* = 0.02 and *p* = 0.002). In taxon-based analysis, stool of CA cases was depleted in a network of *Clostridia* operational taxonomic units from families *Ruminococcaceae*, *Clostridiaceae*, and *Lachnospiraceae*, and enriched in the classes *Bacilli* and *Gammaproteobacteria*, order *Enterobacteriales*, and genera *Actinomyces* and *Streptococcus* (all *q* < 0.10). SSA and HP cases did not differ in diversity or composition from controls, though sample size for these groups was small. Few taxa were differentially abundant between HP cases or SSA cases and controls; among them, class *Erysipelotrichi* was depleted in SSA cases.

**Conclusions:**

Our results indicate that gut microbes may play a role in the early stages of colorectal carcinogenesis through the development of CAs. Findings may have implications for developing colorectal cancer prevention therapies targeting early microbial drivers of colorectal carcinogenesis.

**Electronic supplementary material:**

The online version of this article (doi:10.1186/s40168-016-0218-6) contains supplementary material, which is available to authorized users.

## Background

Colorectal cancer (CRC) is the third most common cancer and fourth most common cause of cancer death worldwide [1]. CRC represents a heterogeneous group of cancers arising through different combinations of genetic and epigenetic events [2]: the “conventional” pathway to CRC is characterized by adenomatous polyposis coli (APC) mutation, chromosomal instability, and paucity of CpG island hypermethylation, while the “serrated” pathway is characterized by B-Raf proto-oncogene, serine/threonine kinase (BRAF) mutation, chromosomal stability, and high CpG island hypermethylation [3]. The majority of CRC cases (~60%) arise via the “conventional” pathway, with ~20% arising from the “serrated” pathway and ~20% arising from an alternate pathway [4]. These distinct molecular pathways originate with different precursor lesions: the “conventional” pathway with conventional adenomas (CAs) and the “serrated” pathway with sessile serrated adenomas (SSAs) [4]. An additional serrated polyp type, the hyperplastic polyp (HP), has negligible malignant potential [2]. Different polyp types also have tendencies to present in specific colorectal locations [2, 5].

Mounting evidence implicates gut bacteria as causal players in colorectal carcinogenesis [6], though their distinct contributions through CAs or SSAs have not been examined simultaneously. Stool transplant experiments from colon tumor-bearing mice or human CRC patients to germ-free mice have revealed a critical role of the gut microbiota in CRC development [7, 8]. Additionally, studies in humans, including a study by our group [9], have associated mucosal or stool microbiota composition with presence of colorectal polyps or CRC [6]. Recently, greater attention has been focused on characterizing the gut microbiota across different stages of colorectal carcinogenesis [10, 11], to better distinguish bacteria contributing to CRC initiation (“driver” bacteria) from bacteria proliferating as a result of CRC (“passenger” bacteria) [12]. Microbes and their metabolites have been proposed to promote carcinogenesis by several mechanisms, including induction of inflammatory signaling pathways, genetic mutations, and epigenetic dysregulation [13–15]. Because CRC arises along different molecular pathways from specific precursor lesions at specific colorectal sites, it is possible that different bacteria are involved in each pathway and associated with each precursor type and/or location; however, no studies have characterized the gut microbiota of colorectal polyp cases according to histologic type and location.

Here, we characterize the microbiota of stool samples from 540 colonoscopy-screened individuals. Detailed endoscopy and pathology reports allowed us to classify these individuals as polyp-free controls, CA cases, HP cases, or SSA cases and to define polyp location within the colorectum. We aimed to determine whether overall microbial community composition differs between these groups and to identify bacterial taxa differing across the groups.

## Methods

### Study population

We included samples from two independent study populations: the Centers for Disease Control and Prevention (CDC) Study of In-home Tests for Colorectal Cancer (SIT), hereafter referred to as the CDC study, and the New York University (NYU) Human Microbiome and Colorectal Tumor study, hereafter referred to as the NYU study.

The CDC study enrolled 451 participants at the University of Minnesota/Minnesota Gastroenterology between December 2012 and July 2014, as part of a study to evaluate the performance of in-home screening tests for CRC. The study participants completed fecal occult blood tests (FOBT) and subsequently underwent colonoscopy. Eligible participants were individuals 50–75 years old scheduled to have a colonoscopy for routine screening only, able to read English, and not currently taking anticoagulant medication. Additionally, participants must not have had more than one episode of rectal bleeding in the last 6 months, a positive FOBT in the past 12 months, a colonoscopy in the past 5 years, a personal history of CRC, polyps, or inflammatory bowel disease, or a personal or family history of familial adenomatous polyposis or hereditary nonpolyposis colorectal cancer. From the 451 subjects, we further excluded 17 who withdrew from the study, 4 subjects for whom sequencing failed, and 32 subjects with both conventional and serrated polyp types or unclassified polyps, resulting in 398 subjects. The CDC study was approved by the institutional review boards (IRB) of the University of Minnesota and the CDC, and all participants provided written consent.

The NYU study enrolled 239 participants from Kips Bay Endoscopy Center in New York City between June 2012 and August 2014. Eligible participants were individuals 18 years or older who recently underwent a colonoscopy, were able to read English, and had not been on long-term antibiotic treatment. We further excluded participants that had missing colonoscopy reports (*n* = 2), personal history of CRC (*n* = 10) or polyps (*n* = 49), inflammatory bowel disease (*n* = 22), previous anastomosis (*n* = 6), personal history of familial adenomatous polyposis (*n* = 1), those with their most recent colonoscopy reports >3 years prior to stool sample collection (*n* = 12), and subjects with both conventional and serrated polyp types or unclassified polyps (*n* = 12); exclusion due to these non-mutually exclusive criteria resulted in 142 subjects remaining. Of these subjects, 54% were receiving a colonoscopy for routine screening, while the remaining 46% had indications for colonoscopy including abdominal pain, rectal bleeding, change in bowel habit, or family history of polyps/cancer. The NYU study was approved by the IRB of NYU School of Medicine, and all participants provided written consent.

### Demographic information assessment

Demographic information (e.g., age, sex, height, weight) was collected by questionnaire in the CDC and NYU studies. BMI was categorized as underweight or normal weight (BMI <25 kg/m^2^), overweight (25 ≤ BMI < 30 kg/m^2^), or obese (BMI ≥30 kg/m^2^).

### Colonoscopy

Colorectal polyps were identified at colonoscopy and confirmed by pathology. Polyp-free controls were defined as those with no polyps identified during colonoscopy and no previous history of colorectal polyps. Subjects with histologically confirmed normal biopsies were also included in the control group. CA cases were defined as those with at least one tubular or tubulovillous adenoma and no other polyps of hyperplastic, SSA, or unclassified histology. We further classified CAs as non-advanced if they were <1 cm and had no villous tissue and as advanced if they were ≥1 cm and/or contained villous tissue [16]. HP cases were defined as having at least one HP, with no other polyps of tubular, tubulovillous, SSA, or unclassified histology. SSA cases were defined as having at least one SSA, with or without HP(s), and with no other polyps of tubular, tubulovillous, or unclassified histology. Proximal polyps were defined as polyps located in the cecum, ascending colon, hepatic flexure, transverse colon, or splenic flexure, and distal polyps were defined as polyps located in the descending colon, sigmoid colon, or rectum. We classified participants as either proximal or distal cases based on the location of their polyp(s); participants with both proximal and distal polyps were classified as distal cases.

### Stool samples

All subjects collected stool samples onto the two sections of Beckman Coulter Hemoccult II SENSA® cards (Beckman Coulter, CA) at home. We have previously shown that sample collection by this method preserves stool microbiota composition assessed by 16S rRNA gene sequencing [17]. Other studies have since confirmed this finding, observing that stool collection card sampling produces reproducible and accurate 16S rRNA gene-derived microbiota data [18] and exhibits stability at room temperature for up to 8 weeks [19]. Samples were collected up to 4 months prior to colonoscopy (range 3–122 days prior) in the CDC study or up to 3 years after colonoscopy (range 5–1026 days after) in the NYU study. CDC participant samples were mailed to a laboratory for fecal occult blood testing within several days of stool collection; this testing does not impact stool microbiota composition [18] (see the Quality control section). After testing, samples were refrigerated at 4 °C until shipment to NYU and, upon arrival, were stored at −80 °C until analysis (range 7–183 days from sample collection to receipt by NYU). NYU participant samples were mailed directly to NYU following at-home collection and stored immediately at −80 °C until analysis.

### Microbiota assay

DNA was extracted from stool using the PowerLyzer PowerSoil Kit (Mo Bio Laboratory Inc., CA) following the manufacturer’s protocol. Briefly, we cut the two sections from the cards containing the stool sample and placed them into 750 μl bead solution. The fecal material in bead solution was lysed using the Powerlyzer (Mo Bio Laboratory Inc., CA) at 4500 rpm for 45 s. DNA was collected and eluted using silica columns included with the PowerLyzer PowerSoil kit. Barcoded amplicons were generated covering the V4 region of the 16S rRNA gene using the F515/R806 primer pair [20]. The PCR reaction was set up using FastStart High Fidelity PCR system, dNTP pack (Roche, IN) and run as follows: an initial denaturing step at 94 °C for 3 min, followed by 25 cycles of 94 °C for 15 s, 52 °C for 45 s, and 72 °C for 1 min, and then a final extension at 72 °C for 8 min. PCR products were purified using Agencourt AMPure XP (Beckman Coulter Life Sciences, IN) and quantified using the Agilent 4200 TapeStation (Agilent Technologies, CA). Amplicon libraries were pooled at equal molar concentrations and sequenced using a 300-cycle (2 × 151 bp) MiSeq reagent kit on the Illumina MiSeq platform for paired-end sequencing.

### Sequence read processing

Forward and reverse reads were joined using *join_paired_ends.py* in QIIME [21], allowing a minimum base-pair overlap of 10 and a maximum of 20% difference in overlap region. Sequences were demultiplexed, and poor-quality sequences excluded, using the default parameters of QIIME script *split_libraries_fastq.py* [21]. From the 540 stool samples, we obtained 19,255,455 quality-filtered 16S rRNA gene sequence reads. Sequence reads were clustered into de novo operational taxonomic units (OTUs) at 97% identity, and representative sequence reads for each OTU were assigned taxonomy based on fully sequenced microbial genomes (IMG/GG Greengenes), using QIIME *pick_de_novo_otus.py* script [21]. Chimeric sequences (identified using ChimeraSlayer [22]), sequences that failed alignment, and singleton OTUs were removed. The final dataset retained 18,617,524 sequences (mean ± SD = 34,477 ± 19,417 sequence reads/sample) and contained 221,501 OTUs.

### Quality control

All samples underwent DNA extraction and sequencing in the same laboratory, and laboratory personnel were blinded to case/control status. A total of 3 sequencing batches were run: 2 for the CDC samples and 1 for the NYU samples. Quality control samples and negative controls were included across all sequencing batches. DNA from 6 stool sample repeats from 4 volunteers were included in each of 3 sequencing batches (2 CDC, 1 NYU) for a total of 72 quality control samples. In order to mimic the sample workflow of the CDC study, 1/6 of the quality control stool samples were treated with Hemoccult SENSA developer (Beckman Coulter, CA). We calculated intra-class correlation coefficients (ICCs) for the Shannon diversity index and DESeq2-normalized counts [23] of abundant bacterial phyla and genera and found the ICCs to be generally high (Additional file 1: Table S1), indicating high similarity of microbiota profiles within repeated samples from the same volunteer. Additionally, principal coordinate analysis (PCoA) showed clustering of the repeated samples from each volunteer regardless of batch or developer treatment, indicating good reproducibility (Additional file 1: Figure S1). Of 9 negative controls (3 in each batch), 6 had zero sequence reads, 2 had 1 read, and 1 had 21 reads, indicating minimal laboratory contamination.

### α-Diversity

Within-subject microbial diversity (α-diversity) was assessed using species richness and the Shannon diversity index, which were calculated in 500 iterations of rarefied OTU tables of 4000 sequence reads per sample. This sequencing depth was chosen to sufficiently reflect the diversity of the samples (Additional file 1: Figure S2) while retaining the maximum number of participants for the analysis (1 control excluded from this analysis due to sequencing depth = 2088). To compare α-diversity between cases and controls, we modeled richness and Shannon index as outcomes in linear regression, adjusting for age, sex, study, and categorical BMI.

### Sequence read count filtering

The raw counts of 221,501 de novo OTUs were agglomerated to 13 phyla, 28 classes, 51 orders, 103 families, and 256 genera. We then filtered out low-count taxa by including only taxa with at least 2 sequence reads in at least 40 participants, resulting in inclusion of 11 phyla, 20 classes, 24 orders, 51 families, 89 genera, and 2347 OTUs (7 of which were of unassigned taxonomy); this filtered data was used in all downstream analyses described below.

### Microbial community types

The stool samples were clustered into community types, or enterotypes, of similar microbial composition at the OTU level using the Dirichlet multinomial mixture (DMM) model [10, 24], implemented using the “DirichletMultinomial” package in *R*. Fisher’s exact test with Monte Carlo simulations was used to determine differences in community types between cases and controls.

### Distances and PERMANOVA

β-Diversity (between-sample differences) was assessed at the OTU level using unweighted and weighted UniFrac phylogenetic distances [25] and the Jensen-Shannon divergence (JSD). The unweighted UniFrac considers only OTU presence or absence, while the weighted UniFrac and JSD take into account OTU relative abundance. Permutational multivariate analysis of variance (PERMANOVA) [26] of the distance matrices, as implemented in the “vegan” package in *R*, was used to identify whether case/control status explains variation in microbial community composition, adjusting first for study, age, sex, and categorical BMI.

### Differential abundance testing

We used negative binomial generalized linear models, as implemented in the “DESeq2” [23] package in *R*, to test for differentially abundant taxa by case/control status at phylum-genus levels and at OTU level. This method models raw count data with a negative binomial distribution and adjusts internally for “size factors” which normalize for differences in sequencing depth between samples. Models were adjusted for sex, age, categorical BMI, and study. DESeq2 default outlier replacement, independent filtering of low-count taxa, and filtering of count outliers were turned off. Taxa models with maximum Cook’s distance >10 were removed prior to *p* value adjustment for the false discovery rate (FDR) [27]. We considered an FDR-adjusted *p* value (*q* value) less than 0.10 as significant.

### OTU correlation network

Spearman’s correlation was used to assess relationships between OTUs that were associated with case/control status. OTU counts were normalized for DESeq2 [23] size factors, to account for differences in library size in a consistent manner to our differential abundance analysis, prior to correlation analysis. Correlations were calculated independently for the groups under comparison (e.g., in control + CA samples). Correlation coefficients with magnitude ≥0.3 were selected for visualization using the “igraph” package in *R*.

## Results

### Participant characteristics

We included a total of 540 colonoscopy-screened individuals in the current analysis, composed of 323 polyp-free controls, 144 cases with CAs only, 40 cases with HPs only, and 33 cases with SSAs (with or without HPs). CA cases were more likely to be male and tended to be older than controls (Table 1). HP cases also tended to be older than controls, while SSA cases did not differ from controls in sex ratio or age. Of the CAs, 15% (*n* = 22) were considered advanced and 38% (*n* = 55) had polyps in the distal colon (Table 1). As expected, the majority of HPs were located in the distal colon (*n* = 34; 85%) and the majority of SSAs were located in the proximal colon (*n* = 30; 91%) (Additional file 1: Table S2).

**Table 1:** Demographic and polyp characteristics of the study participants

	Controls	CA cases	HP cases	SSA cases
*N*	323	144	40	33
Male, %	47.1	67.4**	52.5	54.5
Age (years), mean ± SD	61.3 ± 7.2	63.1 ± 6.6*	64.4 ± 7.5*	63.1 ± 7.0
White^a^, %	94.4	95.0	92.5	97.0
Family history of cancer^b^, %	25.2	29.1	41.0	25.0
BMI category^c^, %				
Under or normal-weight (BMI <25 kg/m^2^)	39.9	31.2	32.5	24.2
Overweight (25 ≤ BMI < 30 kg/m^2^)	38.7	43.1	42.5	45.5
Obese (BMI ≥30 kg/m^2^)	21.4	25.7	25.0	30.3
Study, %				
CDC	75.5	70.1	60.0	87.9
NYU	24.5	29.9	40.0	12.1
Polyp histology^d^, %				
TA <1 cm only		84.0		
TA ≥1 cm, TVA, or TA and TVA only		15.3		
Hyperplastic only			100.0	
SSA only				81.8
SSA and hyperplastic only				18.2
Polyp location^e^, %				
Proximal		60.4	15.0	90.9
Distal		38.2	85.0	9.1

*CA* conventional adenoma, *HP* hyperplastic polyp, *SSA* sessile serrated adenoma

*p < 0.05, **p < 0.001, different from controls by Wilcoxon rank-sum test or Chi-squared test for continuous or categorical variables, respectively

^a^ *n* = 4 were missing race

^b^ *n* = 7 were missing family history

^c^Those missing BMI (CDC *n* = 1, NYU *n* = 3) were re-coded as the median (CDC 27 kg/m^2^, NYU 25 kg/m^2^) in order to retain sample size in covariate adjusted analyses

^d^ *TA* tubular adenoma, *TVA* tubulovillous adenoma, *SSA* sessile serrated adenoma, *n* = 1 subject with a TA could not be classified by size, so conventional adenoma percentage will not sum to 100%

^e^Proximal: polyps only in the cecum, ascending colon, hepatic flexure, transverse colon, or splenic flexure; distal: any polyp located in the descending colon, sigmoid colon, or rectum; see Additional file 1: Table S2 for further breakdown by specific location; *n* = 2 subjects with CAs could not be classified by location, so CA percentage will not sum to 100%

### Global gut microbiota shifts in relation to colorectal polyps

**Fig. 1:**
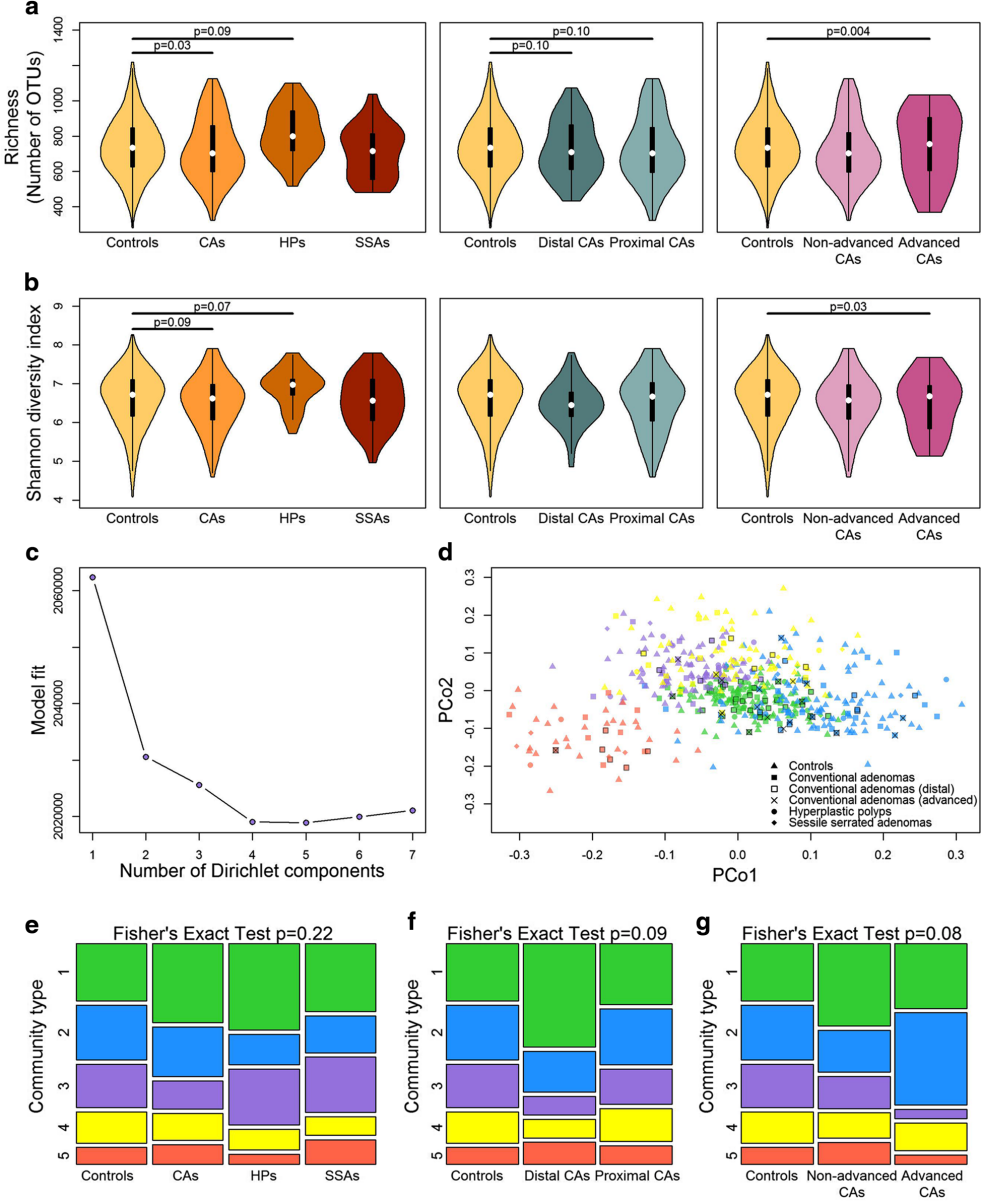
α-Diversity and community types of colonoscopy-screened participants. **a** Violin plots of species richness and **b** Shannon diversity index by polyp histology (controls *n* = 322, CA cases *n* = 144, HP cases *n* = 40, SSA cases *n* = 33), location (distal CA *n* = 55, proximal CA *n* = 87), and advancement level (non-advanced CA *n* = 121, advanced CA *n* = 22). These indices were calculated for 500 iterations of rarefied (4000 sequences per sample) OTU tables, and the average over the iterations was taken for each participant (1 control excluded due to sequencing depth = 2088). *p* values from multiple linear regression are shown. **c** Fitting to the DMM [24, 56] model indicates optimal classification into 5 community types. **d** Principal coordinate analysis of Jensen-Shannon divergence values between participants, colored by community type. *Green* community type 1, *blue* type 2, *purple* type 3, *yellow* type 4, *red* type 5. **e** Distribution of the community types in groups distinguished by histology, **f** location, or **g** advancement level. *p* value from Fisher’s exact test with Monte Carlo simulation is shown. *CAs* conventional adenomas, *HPs* hyperplastic polyps, *SSAs* sessile serrated adenomas

We first investigated microbial community diversity of the participants according to polyp histology and location. CA cases tended to have lower community diversity than controls (richness: *p* = 0.03; Shannon index: *p* = 0.09), a pattern that was consistent for both proximal and distal CA cases, and particularly apparent in advanced CA cases (richness: *p* = 0.004; Shannon index: *p* = 0.03) (Fig. 1a, b; Additional file 1: Table S3). Conversely, HP cases had marginally higher diversity than controls (richness: *p* = 0.09; Shannon index: *p* = 0.07), while community diversity of SSA cases did not differ from controls (richness: *p* = 0.96; Shannon index: *p* = 0.89), though sample sizes for HP and SSA groups were small.

We identified 5 microbial community types among the participants using Dirichlet multinomial mixture models [24] (Fig. 1c, d), each containing controls, CA cases, HP cases, and SSA cases. The top 20 OTUs contributing the most to the Dirichlet components are shown in Additional file 1: Figure S3; OTUs from *Prevotella copri* (increased normalized abundance in community type 5), *Faecalibacterium prausnitzii* (lower normalized abundance in community type 2), and an unclassified *Bacteroides* species (increased normalized abundance in community type 1) were the highest contributors. While the distribution of these community types did not differ significantly by histology (Fig. 1e; Fisher’s exact test *p* = 0.22), we observed a marginally significant difference in community-type distribution by CA polyp location (Fig. 1f; Fisher’s exact test *p* = 0.09) and by CA non-advanced or advanced classification (Fig. 1g; Fisher’s exact test *p* = 0.08). Compared with controls, a higher percentage of distal CA cases were members of community type 1 and fewer were members of community types 3 and 4, while a higher percentage of advanced CA cases were members of community type 2 and fewer were members of community types 3 and 5. Direct comparison of distal CA cases to controls revealed a significant difference in community type distribution between the two groups (Fisher’s exact test *p* = 0.01), though direct comparison of advanced CA cases to controls did not (*p* = 0.20).

PERMANOVA analyses of between-sample distances adjusting for covariates largely supported the findings from the community-type analyses: stool microbial composition of distal CA cases and advanced CA cases tended to differ from controls (distal vs. controls: unweighted UniFrac *p* = 0.02, weighted UniFrac *p* = 0.05, JSD *p* = 0.11; advanced vs. controls: unweighted UniFrac *p* = 0.002, weighted UniFrac *p* = 0.02, JSD *p* = 0.02), while the other case groupings (all CA cases, proximal CA cases, non-advanced CA cases, HP cases, and SSA cases) did not differ significantly from controls (all *p* ≥ 0.10). We did not further classify CA cases into joint location × advanced categories due to sample size restrictions (*n =* 7 in the distal advanced group).

### Taxa associated with conventional adenomas

**Fig. 2:**
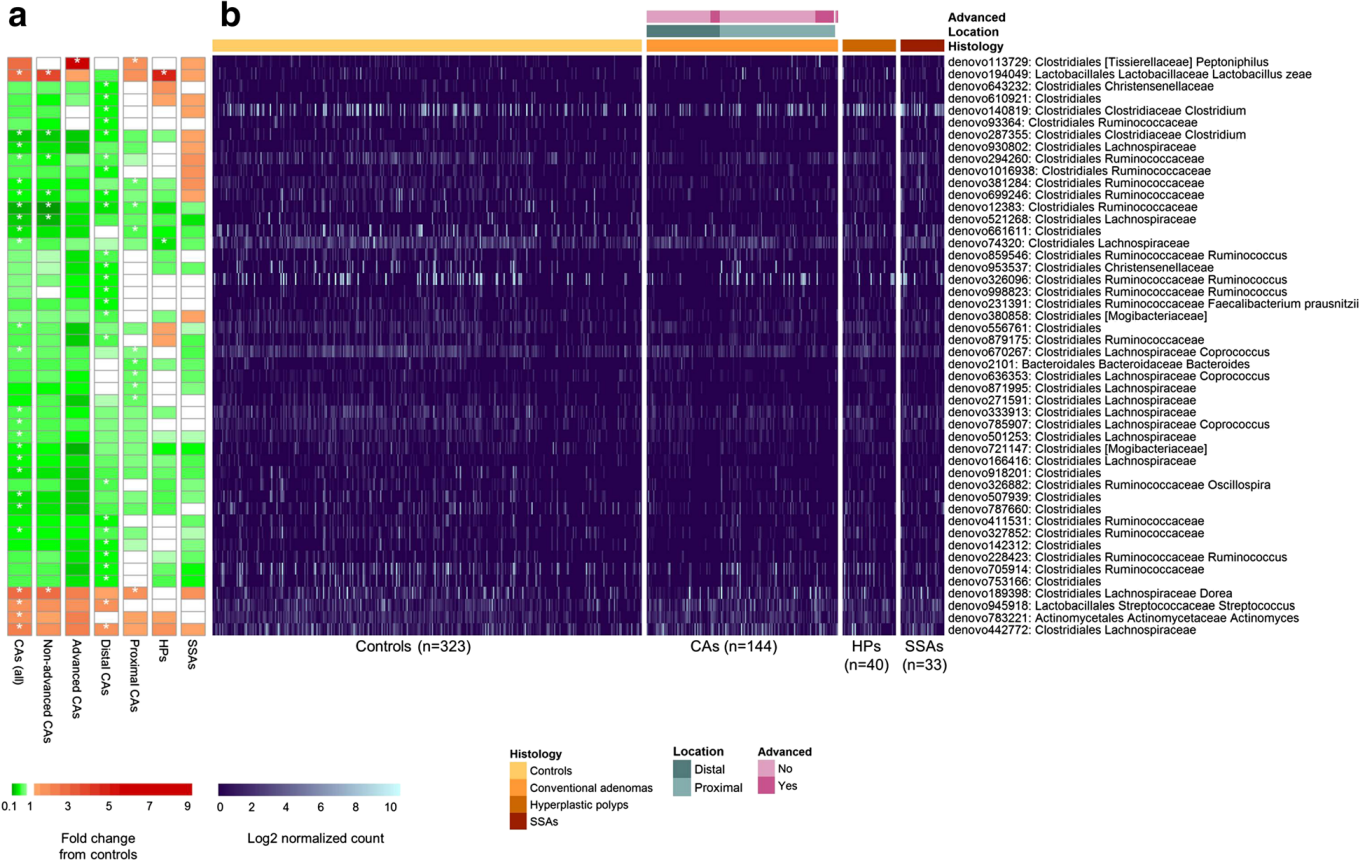
Heatmaps of OTUs that were differentially abundant between colorectal polyp cases and controls. All OTUs with *q* < 0.10 for comparisons of any case group (all CA, non-advanced CA, advanced CA, distal CA, proximal CA, HP, SSA) vs. controls are included in the figure. **a** Heatmap shows fold change from controls in the DESeq2 models, with *white star* indicating *q* < 0.10 for the comparison. **b** Heatmap shows OTU counts in each participant. For display, counts were normalized for DESeq2 size factors and log2 transformed after adding a pseudocount of 1. *n* = 1 and *n* = 2 CA cases were missing advanced status or location information, respectively. *CAs* conventional adenomas, *HPs* hyperplastic polyps, *SSAs* sessile serrated adenomas

**Fig. 3:**
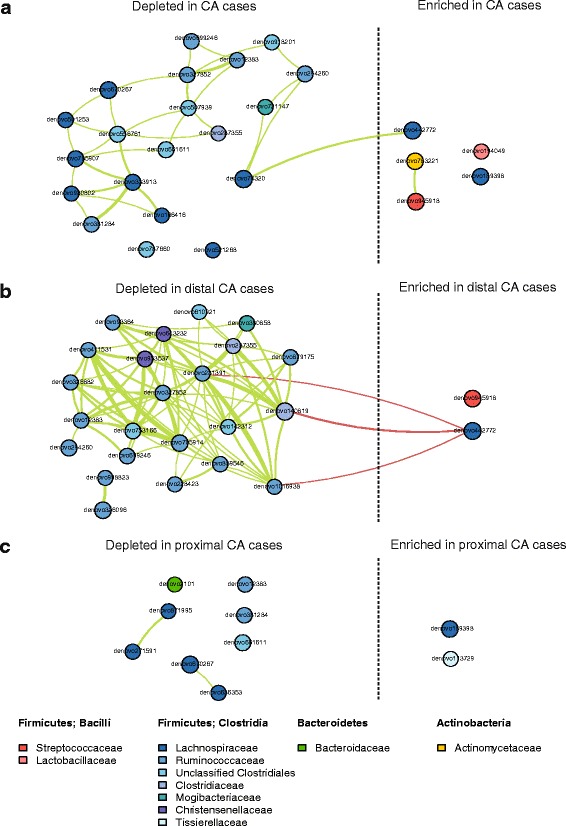
Microbial community ecology in controls and conventional adenoma cases. Correlation network of OTUs differentially abundant between **a** controls and all CA cases, **b** controls and distal CA cases, and **c** controls and proximal CA cases. Spearman’s correlation coefficients were estimated using counts (normalized for DESeq2 size factors) and calculated among the samples under comparison. *Lines* shown between OTUs indicate Spearman’s correlation ≥0.3 (*green*) or ≤-0.3 (*red*). Direction of enrichment in relation to all CA/distal CA/proximal CA cases vs. controls was determined from DESeq2 models. OTUs are colored according to family membership. *Line thickness* represents strength of the correlation, in steps of 0.3–0.4 (*thinnest*), 0.4–0.5, 0.5–0.6, 0.6–0.7, and >0.7 (*thickest*). *CA* conventional adenoma

We next explored taxonomic signatures of the gut microbiota by polyp histology and location using negative binomial generalized linear models [23]. We identified 25 OTUs that were differentially abundant (*q* < 0.10) between CA cases and controls (Fig. 2; Additional file 1: Table S4); 20 of these, all from class *Clostridia*, had decreased normalized abundance in CA cases compared to controls. Conversely, 1 OTU each from *Actinomyces*, *Streptococcus*, *Lactobacillus zeae*, *Dorea*, and an unclassified *Lachnospiraceae* genus had increased normalized abundance in CA cases. Many of the decreased *Clostridia* OTUs formed a correlation network, while the increased *Actinomyces* and *Streptococcus* OTUs were also inter-correlated (Fig. 3a). At broader levels of taxonomic classification, the observed OTU level associations manifested in an observed increased normalized abundance of class *Bacilli* and genera *Streptococcus*, *Actinomyces*, and *Dorea* in CA cases compared to controls (Table 2). Analysis of broader taxonomic classification levels also revealed that CA cases exhibited greater normalized abundance than controls of class *Gammaproteobacteria*, its order *Enterobacteriales*, and genera *Corynebacterium* (class *Actinobacteria*), *Peptoniphilus*, and *Phascolarctobacterium* (class *Clostridia*), and decreased normalized abundance of genus *Coprobacillus* (class *Erysipelotrichi*), and unknown genera within family *Mogibacteriaceae* (class *Clostridia*) and order *RF39* (class *Mollicutes*) (Table 2; Additional file 1: Table S5).

**Table 2:** Differentially abundant^a^ classes and genera between controls and CA cases, HP cases, and SSA cases

		CA cases vs. controls		HP cases vs. controls		SSA cases vs. controls	
Taxon	Mean count^b^	Fold change (95% CI)	*q* ^c^	Fold change (95% CI)	*q* ^c^	Fold change (95% CI)	*q* ^c^
Class							
Firmicutes; Bacilli	506.3	2.11 (1.6, 2.78)	2.45E−06	1.23 (0.78, 1.94)	1.00	0.79 (0.49, 1.3)	0.79
Firmicutes; Erysipelotrichi	305.8	0.92 (0.79, 1.07)	0.57	1.1 (0.85, 1.41)	1.00	0.68 (0.52, 0.89)	0.09
Proteobacteria; Gammaproteobacteria	394.4	1.81 (1.19, 2.76)	0.04	0.32 (0.16, 0.63)	0.02	0.43 (0.21, 0.88)	0.18
Genus							
Actinobacteria; Actinobacteria; Actinomycetales; Actinomycetaceae; Actinomyces	9.1	1.69 (1.32, 2.17)	0.0006	1.24 (0.82, 1.87)	0.99	1.07 (0.7, 1.64)	0.98
Actinobacteria; Actinobacteria; Actinomycetales; Corynebacteriaceae; Corynebacterium	1.0	3.73 (1.88, 7.4)	0.002	0.78 (0.28, 2.22)	0.99	0.86 (0.3, 2.5)	0.98
Firmicutes; Bacilli; Lactobacillales; Streptococcaceae; Streptococcus	422.2	2.38 (1.76, 3.21)	1.35E−06	1.26 (0.78, 2.05)	0.99	0.72 (0.43, 1.21)	0.65
Firmicutes; Clostridia; Clostridiales; NA; NA^d^	2624.0	1.44 (1.26, 1.65)	6.31E−06	1.18 (0.94, 1.47)	0.92	0.98 (0.77, 1.25)	0.98
Firmicutes; Clostridia; Clostridiales; [Mogibacteriaceae]; NA^d^	16.2	0.78 (0.65, 0.94)	0.06	0.85 (0.63, 1.15)	0.99	0.89 (0.65, 1.22)	0.88
Firmicutes; Clostridia; Clostridiales; [Tissierellaceae]; Peptoniphilus	1.6	3.41 (1.83, 6.35)	0.002	0.55 (0.21, 1.46)	0.99	0.85 (0.32, 2.28)	0.98
Firmicutes; Clostridia; Clostridiales; Lachnospiraceae; Anaerostipes	45.7	1.23 (0.98, 1.54)	0.30	1.96 (1.36, 2.84)	0.03	1.02 (0.69, 1.52)	0.98
Firmicutes; Clostridia; Clostridiales; Lachnospiraceae; Dorea	216.1	1.19 (1.05, 1.35)	0.06	1.09 (0.88, 1.34)	0.99	1 (0.8, 1.25)	0.99
Firmicutes; Clostridia; Clostridiales; Veillonellaceae; Phascolarctobacterium	115.4	1.91 (1.24, 2.96)	0.04	1.12 (0.57, 2.22)	0.99	0.85 (0.41, 1.76)	0.98
Firmicutes; Erysipelotrichi; Erysipelotrichales; Erysipelotrichaceae; Coprobacillus	10.8	0.59 (0.39, 0.88)	0.07	0.36 (0.19, 0.68)	0.07	1.03 (0.53, 2.01)	0.98
Proteobacteria; Betaproteobacteria; Burkholderiales; Alcaligenaceae; Sutterella	430.2	2.06 (1.52, 2.78)	8.60E−05	0.84 (0.52, 1.37)	0.99	0.97 (0.58, 1.65)	0.98
Tenericutes; Mollicutes; RF39; NA; NA^d^	36.4	0.42 (0.22, 0.78)	0.06	1.1 (0.44, 2.77)	0.99	1.06 (0.4, 2.81)	0.98

*CA*, conventional adenoma, *HP* hyperplastic polyp, *SSA* sessile serrated adenoma

^a^Differential abundance was detected by the “DESeq” function in the DESeq2 package. All classes and genera with an FDR-adjusted *q* < 0.10 are included in the table. Models were adjusted for sex, age, study, and categorical BMI. See Additional file 1: Table S5 for comparisons at the phylum, order, and family level

^b^Counts were normalized by dividing raw counts by DESeq2 size factors

^c^FDR-adjusted *p* value. FDR adjustment was conducted at each level (i.e., class, genus) separately

^d^NA: unclassified genus

CAs were further classified as proximal (*n* = 87) or distal (*n* = 55) and as non-advanced (*n* = 121) or advanced (*n* = 22), in order to explore taxonomic signatures associated with these sub-groups. Many OTUs from class *Clostridia* had decreased normalized abundance in distal CA cases compared to controls, including OTUs from families *Ruminococcaceae*, *Clostridiaceae*, *Christensenellaceae*, and *Mogibacteriaceae* (Fig. 2; Additional file 1: Table S6). These OTUs formed a positive correlation network with each other (Fig. 3b). One OTU from *Streptococcus* and one from *Lachnospiraceae* had increased normalized abundance in distal CA cases, and the OTU from *Lachnospiraceae* was inversely correlated with several of the decreased *Clostridia* OTUs (Fig. 3b). These OTU level associations manifested in associations at broader taxonomic levels, including significantly decreased normalized abundance of class *Clostridia* and families *Mogibacteriaceae*, *Christensenellaceae*, and *Clostridiaceae* in distal CA cases compared to controls (Additional file 1: Table S6). Proximal CA cases also had some differentially abundant OTUs from controls, including *Dorea* and *Peptoniphilus* OTUs (increased normalized abundance in proximal CA cases), and *Bacteroides*, *Coprococcus*, and unclassified *Lachnospiraceae* and *Ruminococcaceae* OTUs (decreased normalized abundance in proximal CA cases) (Fig. 2; Additional file 1: Table S6); most of these OTUs were uncorrelated with one another (Fig. 3c). Analysis at broader levels of taxonomic classification revealed additional differences between proximal CA cases and controls that were not all apparent at the OTU level; similar to the all CA case analysis, proximal CA cases exhibited greater normalized abundance than controls of classes *Bacilli* and *Gammaproteobacteria*, order *Enterobacteriales*, and genera *Actinomyces*, *Corynebacterium*, *Streptococcus*, *Dorea*, *Peptoniphilus*, and *Phascolarctobacterium*, among others (Additional file 1: Table S6).

Although the overall microbiota composition of advanced CA cases was significantly different from controls in the global analysis, we observed only one differentially abundant OTU (from genus *Peptoniphilus*, *q* < 0.10) between advanced CA cases and controls (Fig. 2; Additional file 1: Table S7); this is likely an issue of low power as the sample size of advanced CA cases was small (*n* = 22). However, both non-advanced and advanced CA cases exhibited similar directions of fold change in OTU normalized abundance from controls (Fig. 2), indicating similarity between the two groups. At broader taxonomic classification levels, advanced CA cases exhibited greater normalized abundance than controls of genera *Actinomyces*, *Corynebacterium*, *Peptoniphilus*, *Porphyromonas*, and *Haemophilus* and lower normalized abundance than controls of genera *Lachnospira*, *Lachnobacterium*, and unclassified genera from *Mogibacteriaceae*, *Christensenellaceae*, and *RF39* (Additional file 1: Table S7). Non-advanced CA cases, making up the majority of all CA cases, exhibited similar differentially abundant taxa from controls as in the all CA case analysis.

### Taxa associated with hyperplastic polyps and SSAs

We identified few differentially abundant taxa between HP cases or SSA cases and controls (*q* < 0.10). HP cases had increased normalized abundance of *Lactobacillus zeae* and decreased normalized abundance of an unidentified OTU in family *Lachnospiraceae* (Fig. 2; Additional file 1: Table S4). HP cases also had decreased normalized abundance of class *Gammaproteobacteria*, order *Enterobacteriales*, and genus *Coprobacillus* and increased normalized abundance of genus *Anaerostipes*, compared to controls (Table 2; Additional file 1: Table S5). SSA cases had decreased normalized abundance of class *Erysipelotrichi* (Table 2); however, no other taxa (phylum-genus levels or OTU level) were identified as differentially abundant (*q* < 0.10) between SSA cases and controls.

### Sensitivity analysis

We conducted our main analysis excluding participants (*n* = 5) who collected their stool sample <2 weeks after their colonoscopy, in order to ensure results were not biased by effects of colon preparation and colonoscopy on the microbiota (Additional file 1: Table S8). We also conducted our main analysis excluding participants who had taken antibiotics within 30 days prior to sample collection (*n* = 19 from the NYU study), in order to ensure results were not biased by effects of antibiotics on the microbiota (antibiotic usage information was not available in the CDC study) (Additional file 1: Table S9). Excluding these participants groups did not substantially impact findings.

## Discussion

In this large study of colonoscopy-screened adults, we found that CA-associated changes in gut microbiota diversity and composition in relation to controls depended on the severity and location of the adenoma. More specifically, advanced CA cases had the greatest reduction in community diversity compared to controls, while distal or advanced CA cases differed significantly in microbiota composition from controls. Such differences were not observed for subjects with hyperplastic polyps or SSAs. Our results indicate that gut bacteria may play distinct roles in the development of site-specific histologically different polyp types. To our knowledge, this is the first study to simultaneously consider different polyp histologies and locations and the largest study of the gut microbiota and colorectal polyps to date.

Our finding of reduced species richness and diversity in CA cases, particularly advanced CA cases, is consistent with observations in CRC from our group in the USA [9] and from another group in China [28]. Decreased gut microbial diversity, often observed in other diseases including obesity [29] and inflammatory bowel diseases [30, 31], is likely indicative of underlying bacterial dysbiosis, possibly due to domination by opportunistic pathogenic bacteria and/or loss of commensal bacteria. While other reports of colorectal polyp [32–35] and cancer [10, 36–38] showed mixed results in regard to community diversity, including findings of no differences in diversity or increased diversity in cases, sample sizes for these studies were small (*N* for cases ranged from 7 to 53). These differing results may be related to limited power or to the specific bacterial drivers or pathogens present in each unique study population.

Our observation of global OTU-level composition shifts in distal, but not proximal, CA cases compared to controls is likely due to stool being a better proxy for the bacterial communities of the distal colon than of the proximal colon [39]. This was proposed in a recent metagenomic study of colorectal cancer, in which carcinoma-associated bacterial genes were more abundant in stool of distal CRC cases than proximal CRC cases [10]. Additionally, there is evidence that mucosal bacterial biofilms play a role in proximal, but not distal, CRCs [40], further suggesting that bacteria are involved in proximal tumor formation, but that stool may be an inappropriate sample to test their involvement. However, despite the lack of global OTU-level shifts in proximal CA cases compared to controls, we did observe a taxonomic signature for proximal CA cases at broader levels of taxonomic classification; further, this signature differed from that of distal CA cases. This finding suggests that the role bacteria play in CA development may differ between proximal and distal colon sites. There are known molecular distinctions between proximal and distal CRCs, most notably that proximal CRCs are more likely to be hypermethylated and to have elevated mutation rates [41]. Additionally, the luminal environment differs between proximal and distal colon sites: there are high levels of easily fermentable carbohydrate substrates in the proximal colon, which decrease distally through the colon [39, 42]; the mucus layer increases in thickness distally through the colon [42]; the number of bacterial cells increases distally through the colon [43]; and immune activity decreases distally through the colon [44]. These differences can result in site-specific bacterial communities and processes, which may contribute to CA development in distinct ways.

A major shift in stool microbiota composition observed for CA cases was the depleted normalized abundance of a network of *Clostridia* OTUs from families *Ruminococcaceae*, *Clostridiaceae*, and *Lachnospiraceae*; this was particularly apparent in distal CA cases, in which the class *Clostridia* was significantly depleted. Members of these *Clostridia* families have in common the capacity to generate butyrate from fermentation of non-digestible plant fibers [45], which is beneficial to colonic health [46]. Depletion of butyrate-producing bacteria in the distal colon, where carbohydrate substrate supply is already compromised [39, 42], may allow for adenoma growth. The decreased normalized abundance of *Clostridia* we have observed here is consistent with our previous study of CRC, in which the relative abundance of class *Clostridia* was depleted in stool samples of CRC cases compared to controls [9]. Other studies have also found decreased relative abundance of butyrate-producing bacteria in adenoma [33, 47, 48] and CRC [36, 37], supporting the protective effects of butyrate against CRC.

The taxonomic signature of proximal CA cases was not apparent at the OTU-level, though distinct patterns emerged at broader levels of taxonomic classification; this is perhaps because the stool microbiota are a poor proxy for the microbiota of the proximal colon, thus reducing power to detect OTU-level differences between proximal CA cases and controls. Proximal CA cases exhibited greater normalized abundance than controls of classes *Bacilli* and *Gammaproteobacteria*, order *Enterobacteriales*, and genera *Actinomyces*, *Corynebacterium*, *Streptococcus*, *Dorea*, *Peptoniphilus*, and *Phascolarctobacterium*; some of these bacteria may be candidate drivers of the CA pathway in the proximal colon. Some results from other studies are similar to these findings (though none of these studies have distinguished adenomas by location): the genera *Dorea* [48], *Phascolarctobacterium* [48], and *Streptococcus* [32, 33, 49], as well as genera within the *Enterobacteriaceae* family of *Gammaproteobacteria* [32, 34, 35, 48, 49], were elevated in mucosal or stool samples from adenoma cases compared to controls. Additionally, the *Enterobacteriaceae* family and *Actinomycetales* order have been highlighted as potential CRC driver bacteria, based on their over-representation in off-tumor compared to on-tumor paired samples from CRC patients [12]. Members of *Enterobacteriaceae* are known to cause inflammation in the gastrointestinal tract and could contribute to CRC via inflammatory mechanisms [12, 50]. Interestingly, a recent report on CRCs found that invasive polymicrobial bacterial biofilms were a key feature of proximal colon tumors, but not distal tumors [40]. This study implicated the organization, rather than composition, of mucosal communities in proximal CRC development. It will be important for future studies to examine the mucosal communities of proximal CAs and to determine the microbial organizational and/or compositional factors associated with their presence.

The observation that the stool microbial composition of SSA cases was similar to that of controls was unexpected, since an animal model [51] and human study [52] suggest involvement of host microbiota in serrated polyp development. We did observe a decrease in the *Erysipelotrichi* class in SSA cases; this class has been associated with colon mucus barrier impenetrability in mice [53] and may play a protective role in SSA development. Our lack of other findings is likely related to low power due to the small sample size of SSA cases and the proximal location of SSAs. Another potential explanation for this finding is the possibility that bacteria may initiate CRC via a mechanism related to the conventional pathway, but not serrated pathway, such as by inducing chromosomal instability [54].

Strengths of this study include the large sample size, the histologic and location classification of polyps for all cases, the inclusion of polyp-free controls, and the comprehensive bacterial profiling using 16S rRNA gene sequencing. However, this study also has several limitations. We did not examine colorectal mucosal samples; while easily obtainable stool samples are important for developing tools for risk stratification and screening for CRC [38, 47], mucosal samples are important from a prevention standpoint, as they allow for better identification of bacteria associated with adenoma. Assessment of differences in the stool microbiota between polyp cases and polyp-free controls may provide insight into systematic differences in the gut microbiota between these groups that may contribute to polyp development. Future studies incorporating mucosal samples will be able to better pinpoint specific mucosal-associated bacteria responsible for polyp initiation and growth. Further limitations are the mostly white study population, limiting generalizability to other racial groups, the lack of antibiotic usage information in the CDC study, and the cross-sectional design, which does not allow us to establish the temporality of the bacteria-adenoma relationship.

## Conclusions

Due to the different molecular origins and etiologies of CRC, which may vary by colon site [44], it is critically important to consider that the role bacteria play in adenoma development may differ by polyp histology and location, as our results suggest. Although evidence is mounting for a role of driver bacteria in colorectal carcinogenesis, it is likely that different bacterial drivers can confer the same risk for CRC [12]. Bacterial drivers may differ between patients and populations and between polyp histologies and locations. The possibility that there are multiple population-specific, histology-specific, and site-specific bacterial drivers of CRC highlights the need for additional, larger studies in different populations, taking into consideration polyp histology and location, in order to fully characterize the broad array of potential bacterial drivers of CRC, as well as potential protective bacteria, and to identify their functions. Identification of the bacterial drivers of CRC may lead to development of targeted prophylactic therapies. Identification of beneficial bacteria depleted in adenomas may lead to implementation of dietary interventions or probiotic/prebiotic therapies to promote their regrowth and recolonization [55]. Thus, continued study of the early stages of the adenoma-carcinoma sequence may lead to actionable means for CRC prevention.

## Additional file

Additional file 1: Figures S1-S3 and Tables S1-S9.
**Figure S1.** Principal coordinate analysis (PCoA) of the unweighted and weighted UniFrac distances for quality control stool specimens. **Figure S2.** Rarefaction curves of richness and the Shannon index. **Figure S3.** Count heatmap of top 20 OTUs contributing the most to the Dirichlet components of the Dirichlet multinomial mixture model. **Table S1.** Quality control intra‐class correlation coefficients (ICCs) and 95% CIs for the Shannon index and normalized counts of selected phyla and genera. **Table S2.** Number of participants with polyp(s) in the specified colon locations, stratified by assignment into case type and polyp location groupings used in analysis. **Table S3.** Richness and Shannon diversity index by group. **Table S4.** Differentially abundant OTUs between controls and conventional adenoma cases, hyperplastic polyp cases, or SSA cases. **Table S5.** Differentially abundant taxa (phylum‐genus levels) between controls and conventional adenoma cases, hyperplastic polyp cases, or SSA cases.** Table S6.** Differentially abundant taxa (phylum‐OTU level) between controls and proximal or distal conventional adenoma cases. **Table S7.** Differentially abundant taxa (phylum‐OTU level) between controls and non‐advanced or advanced conventional adenoma cases. **Table S8.** Sensitivity analysis—excluding participants (n = 5) who collected their stool sample <2 weeks after their colonoscopy. **Table S9.** Sensitivity analysis—excluding participants (n = 19 from the NYU study) who had taken antibiotics within 30 days prior to sample collection (antibiotic usage information was not available in the CDC study). (PDF 631 kb)
