# Erratum to: The gut microbiota in conventional and serrated precursors of colorectal cancer

**DOI:** 10.1186/s40168-017-0238-x

**Published:** 2017-03-06

**Authors:** Brandilyn A. Peters, Christine Dominianni, Jean A. Shapiro, Timothy R. Church, Jing Wu, George Miller, Elizabeth Yuen, Hal Freiman, Ian Lustbader, James Salik, Charles Friedlander, Richard B. Hayes, Jiyoung Ahn

**Affiliations:** 1 Department of Population Health, New York University School of Medicine, New York, NY USA; 2 Division of Cancer Prevention and Control, Centers for Disease Control and Prevention, Atlanta, GA USA; 3 Division of Environmental Health Sciences, School of Public Health, University of Minnesota, Minneapolis, MN USA; 4 Department of Surgery, New York University School of Medicine, New York, NY USA; 5 Department of Cell Biology, New York University School of Medicine, New York, NY USA; 6 NYU Perlmutter Cancer Center, New York University School of Medicine, New York, NY USA; 7 Kips Bay Endoscopy Center, New York, NY USA

## Erratum

Upon publication of this article [1], it was requested that: “Samples were sequenced at the NYUMC Genome Technology Center. The NYUMC Genome Technology Center is partially supported by the Cancer Center Support Grant, P30CA016087, at the Laura and Isaac Perlmutter Cancer Center” be added to the Acknowledgements section. This acknowledgement has been included by means of this erratum.
