# Supplementary material for: The gut microbiota in conventional and serrated precursors of colorectal cancer
Source: Microbiome. 2016 Dec 30;4:69. doi: 10.1186/s40168-016-0218-6 (PMC5203720; doi:10.1186/s40168-016-0218-6)
Supplement: Additional file 1: Figures S1-S3 and Tables S1-S9. — Figure S1. Principal coordinate analysis (PCoA) of the unweighted and weighted UniFrac distances for quality control stool specimens. Figure S2. Rarefaction curves of richness and the Shannon index. Figure S3. Count heatmap of top 20 OTUs contributing the most to the Dirichlet components of the Dirichlet multinomial mixture model. Table S1. Quality control intra‐class correlation coefficients (ICCs) and 95% CIs for the Shannon index and normalized counts of selected phyla and genera. Table S2. Number of participants with polyp(s) in the specified colon locations, stratified by assignment into case type and polyp location groupings used in analysis. Table S3. Richness and Shannon diversity index by group. Table S4. Differentially abundant OTUs between controls and conventional adenoma cases, hyperplastic polyp cases, or SSA cases. Table S5. Differentially abundant taxa (phylum‐genus levels) between controls and conventional adenoma cases, hyperplastic polyp cases, or SSA cases. Table S6. Differentially abundant taxa (phylum‐OTU level) between controls and proximal or distal conventional adenoma cases. Table S7. Differentially abundant taxa (phylum‐OTU level) between controls and non‐advanced or advanced conventional adenoma cases. Table S8. Sensitivity analysis—excluding participants (n = 5) who collected their stool sample <2 weeks after their colonoscopy. Table S9. Sensitivity analysis—excluding participants (n = 19 from the NYU study) who had taken antibiotics within 30 days prior to sample collection (antibiotic usage information was not available in the CDC study). (PDF 631 kb) [file 40168_2016_218_MOESM1_ESM.pdf]

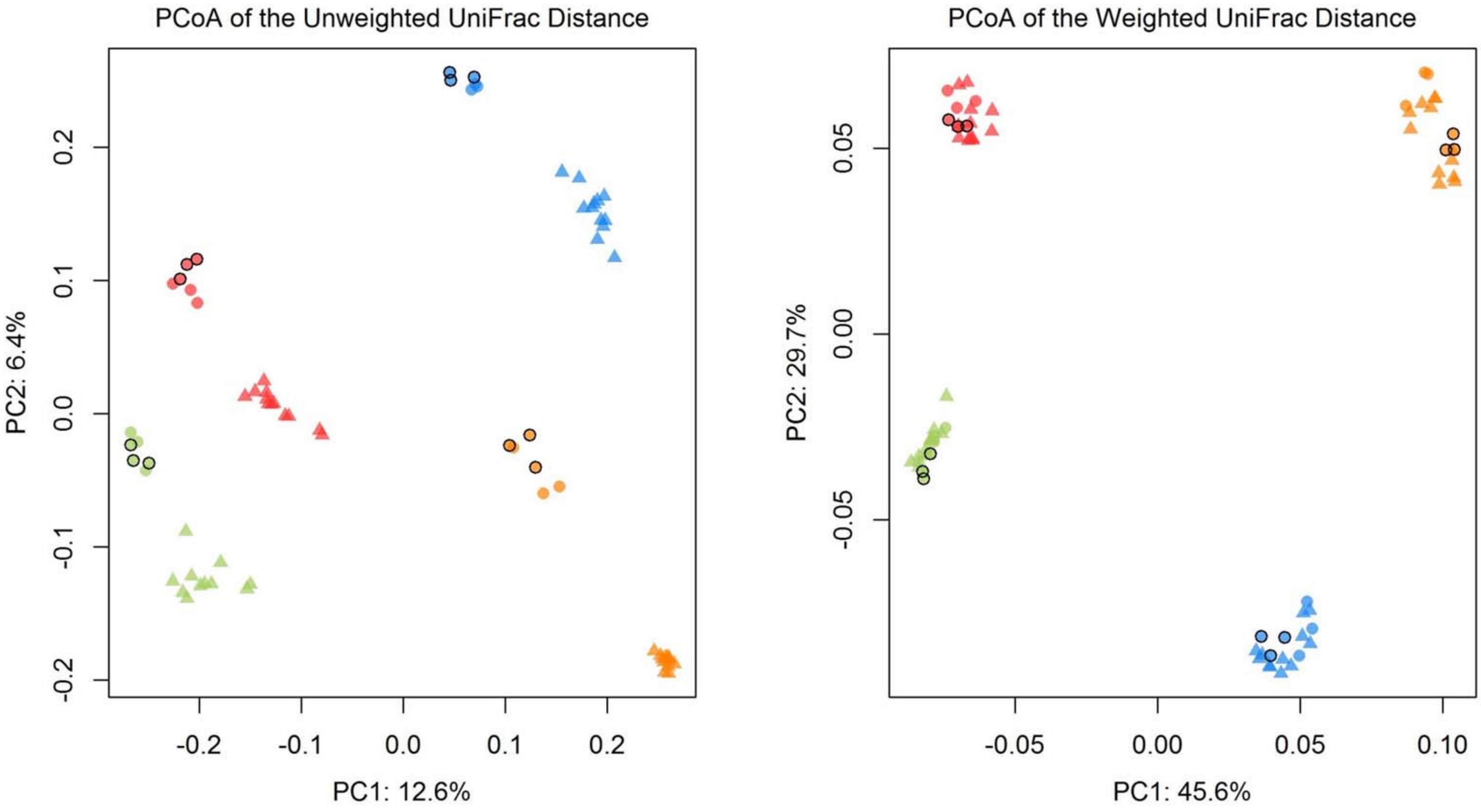

Supplementary Figure 1. Principal coordinate analysis (PCoA) of the unweighted and weighted UniFrac distances for quality control stool specimens provided by 4 volunteers. The 4 volunteers are differentiated by color, with triangles representing the repeats sequenced in the two CDC sequencing batches, and circles representing the repeats sequenced in the NYU sequencing batch. Circles outlined in black represent quality control stool specimens in the NYU batch that were treated with Beckman Coulter Hemoccult SENSA developer.

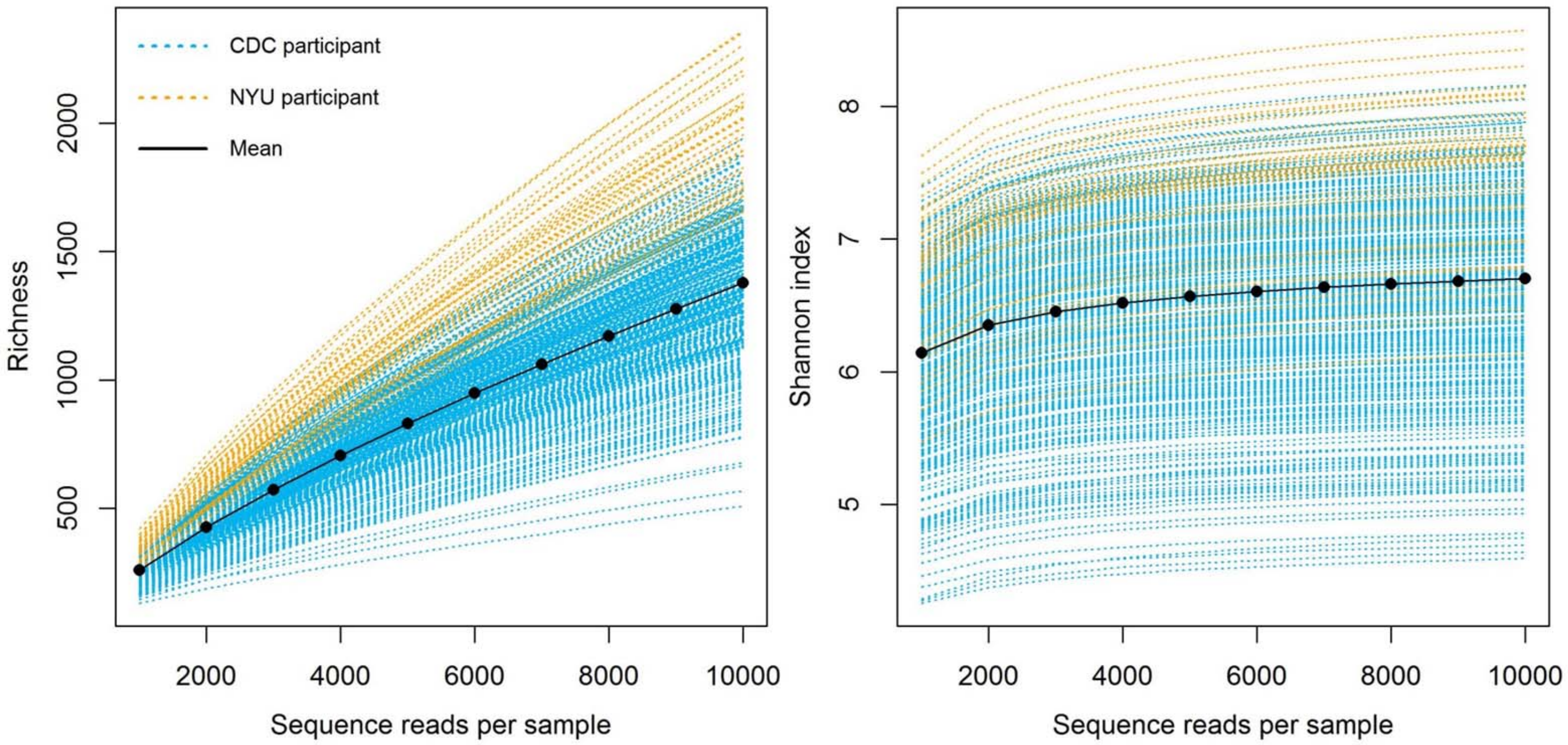

Supplementary Figure 2. Rarefaction curves of richness and the Shannon index. These indices were calculated for 500 iterations of rarefied OTU tables, and the average over the iterations was taken for each participant. n=438 participants with sequencing depths ≥10,000 are plotted.

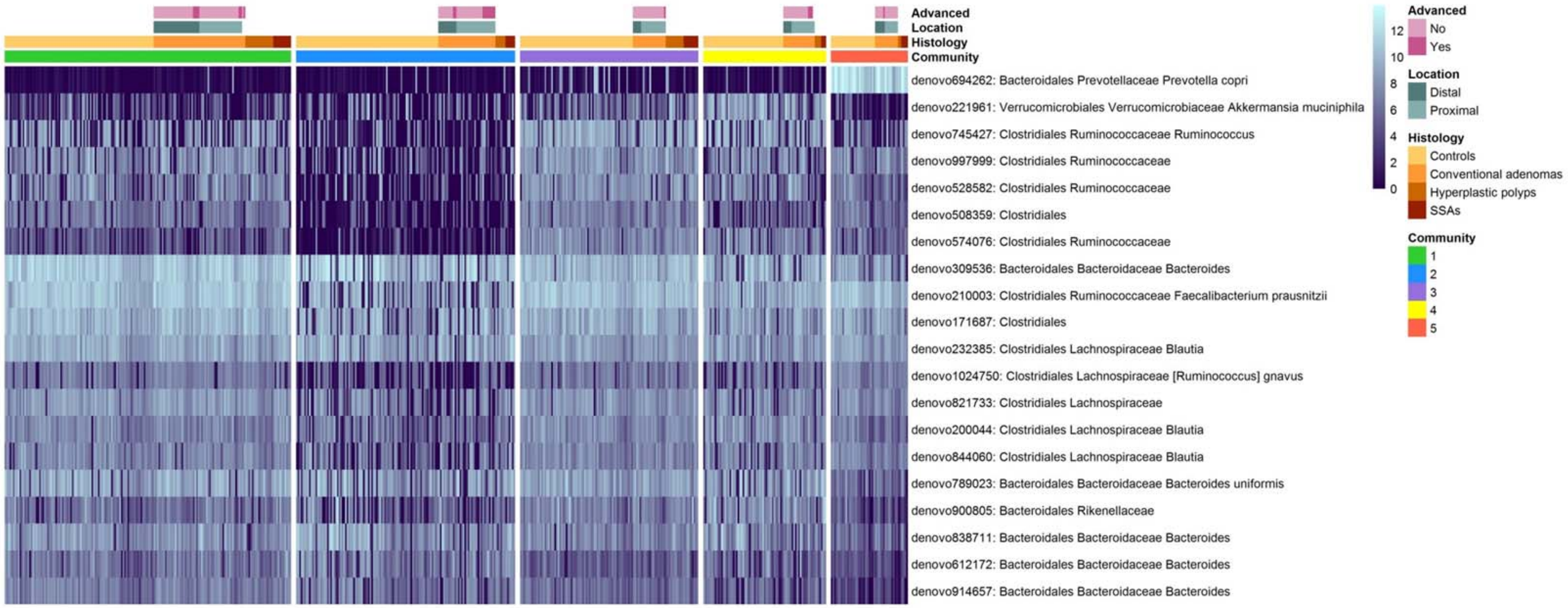

Supplementary Figure 3. Count heatmap of top 20 OTUs contributing the most to the Dirichlet components of the Dirichlet multinomial mixture model. For heatmap display, counts were normalized for DESeq2 size factors and log2 transformed after adding a pseudocount of 1.

Supplementary Table 1. Quality control intra-class correlation coefficients (ICCs) and 95% CIs for the Shannon index and normalized counts of selected phyla and genera. Six repeats from four volunteers were included in each of 3 sequecing batches (2 CDC, 1 NYU) for a total of 72 quality control samples. Counts were normalized by dividing the raw counts by DESeq size factors.

	**CDC**	**NYU**	**All**
Shannon Index	0.98 (0.94, 1)	0.98 (0.93, 1)	0.88 (0.69, 0.99)
Firmicutes	0.7 (0.38, 0.97)	0.61 (0.22, 0.96)	0.66 (0.35, 0.97)
Coprococcus	0.94 (0.81, 1)	0.88 (0.63, 0.99)	0.92 (0.77, 0.99)
Lachnospira	0.99 (0.98, 1)	0.99 (0.97, 1)	0.99 (0.97, 1)
Blautia	0.96 (0.88, 1)	0.97 (0.88, 1)	0.97 (0.89, 1)
Faecalibacterium	0.89 (0.69, 0.99)	0.89 (0.66, 0.99)	0.87 (0.66, 0.99)
Ruminococcus	0.99 (0.97, 1)	1 (0.98, 1)	0.99 (0.97, 1)
Dorea	0.81 (0.54, 0.98)	0.65 (0.27, 0.97)	0.77 (0.49, 0.98)
Bacteroidetes	0.77 (0.48, 0.98)	0.65 (0.27, 0.97)	0.74 (0.45, 0.98)
Bacteroides	0.81 (0.55, 0.98)	0.83 (0.53, 0.99)	0.83 (0.58, 0.99)
Proteobacteria	0.51 (0.19, 0.94)	0.5 (0.12, 0.94)	0.52 (0.22, 0.94)
Sutterella	0.4 (0.12, 0.91)	0.52 (0.14, 0.95)	0.41 (0.15, 0.91)
Actinobacteria	0.92 (0.76, 0.99)	0.85 (0.58, 0.99)	0.87 (0.67, 0.99)
Bifidobacterium	0.94 (0.83, 1)	0.93 (0.78, 1)	0.92 (0.77, 0.99)

Supplementary Table 2. Number of participants with polyp(s) in the specified colon locations, stratified by assignment into case type and polyp location groupings used in analysis. Proximal polyps were defined as polyps located in the cecum, ascending colon, hepatic flexure, transverse colon, or splenic flexure, and distal polyps were defined as polyps located in the descending colon, sigmoid colon, or rectum. We classified participants as either proximal or distal cases based on the location of their polyp(s); participants with both proximal and distal polyps were classified as distal cases. Numbers will sum to greater than group sample sizes due to multiple polyps with differing locations per participant.

	Conventional adenoma cases (n=144)		Hyperplastic polyp cases (n=40)		SSA cases (n=33)	
	Proximal (n=87)	Distal (n=55)	Proximal (n=6)	Distal (n=34)	Proximal (n=30)	Distal (n=3)
Cecum	25	6	0	1	10	0
Ascending colon	38	7	3	0	13	0
Hepatic flexure	9	1	0	0	1	0
Transverse colon	33	5	4	2	7	1
Splenic flexure	3	1	0	1	0	0
Descending colon	0	16	0	2	0	1
Sigmoid colon	0	30	0	23	0	1
Rectum	0	11	0	15	0	1

Supplementary Table 3. Richness and Shannon diversity index by group.

	Richness[a]			Shannon index[a]		
	**Mean (95% CI)**	**B (95% CI)[b]**	**P-value[b]**	**Mean (95% CI)**	**B (95% CI)[b]**	**P-value[b]**
Controls (n=322)	740 (722, 758)	NA	NA	6.6 (6.5, 6.7)	NA	NA
CA cases (n=144)	726 (697, 755)	-28.6 (-53.8, -3.4)	0.03	6.5 (6.4, 6.6)	-0.11 (-0.25, 0.02)	0.09
Proximal (n=87)	726 (687, 765)	-25.6 (-55.9, 4.6)	0.10	6.5 (6.4, 6.7)	-0.09 (-0.25, 0.07)	0.27
Distal (n=55)	725 (682, 768)	-30.7 (-66.9, 5.4)	0.10	6.5 (6.3, 6.6)	-0.14 (-0.33, 0.05)	0.14
Non-advanced (n=121)	721 (690, 752)	-19.0 (-45.8, 7.8)	0.16	6.5 (6.4, 6.6)	-0.08 (-0.22, 0.06)	0.28
Advanced (n=22)	744 (655, 832)	-80.1 (-134.5, -25.6)	0.004	6.5 (6.1, 6.8)	-0.32 (-0.60, -0.03)	0.03
HP cases (n=40)	818 (773, 863)	35.6 (-5.9, 77.2)	0.09	6.9 (6.7, 7.1)	0.20 (-0.01, 0.42)	0.07
SSA cases (n=33)	711 (660, 763)	-1.2 (-46.2, 43.7)	0.96	6.5 (6.3, 6.8)	0.02 (-0.22, 0.25)	0.89

[a]These indices were calculated for 500 iterations of rarefied (4000 sequences per sample) OTU tables, and the average over the iterations was taken for each participant (1 control excluded due to sequencing depth=2088).
[b]Betas and p-values for difference between cases and controls from multiple linear regression, adjusting for age, sex, categorical BMI, and study.

Supplementary Table 4. Differentially abundant OTUs between controls and conventional adenoma cases, hyperplastic polyp cases, or SSA cases, as detected by the DESeq function in the DESeq2 package. Models were adjusted for age, sex, categorical BMI, and study. All OTUs with an FDR-adjusted q<0.10 are included in the table.

					Conventional adenoma cases vs. controls			Hyperplastic polyp cases vs. controls			SSA cases vs. controls		
**Taxon name**	**Taxon classification**	**Level**	**Mean normalized count**	**Maximum Cook's distance**	**Fold change (95% CI)**	**p-value**	**q-value**	**Fold change (95% CI)**	**p-value**	**q-value**	**Fold change (95% CI)**	**p-value**	**q-value**
denovo783221	Actinobacteria; Actinobacteria; Actinomycetales; Actinomycetaceae; Actinomyces; NA	OTU	2.33	0.08	1.54 (1.2, 1.97)	0.0007541	0.0797238	1.33 (0.89, 1.98)	0.1608159	0.9974069	1.01 (0.67, 1.53)	0.9532205	0.998464
denovo194049	Firmicutes; Bacilli; Lactobacillales; Lactobacillaceae; Lactobacillus; zeae	OTU	1.92	6.39	2.93 (1.62, 5.32)	0.0003857	0.0529539	4.65 (2.21, 9.79)	5.35E-05	0.062395	1.36 (0.64, 2.91)	0.4225708	0.9816193
denovo945918	Firmicutes; Bacilli; Lactobacillales; Streptococcaceae; Streptococcus; NA	OTU	4.86	0.07	1.81 (1.31, 2.52)	0.0003834	0.0529539	1 (0.6, 1.65)	0.9928819	0.9982711	0.97 (0.57, 1.64)	0.9111805	0.9969289
denovo721147	Firmicutes; Clostridia; Clostridiales; [Mogibacteriaceae]; NA; NA	OTU	0.82	0.12	0.43 (0.26, 0.69)	0.0004905	0.0602595	0.49 (0.25, 0.95)	0.0343854	0.9974069	0.42 (0.21, 0.83)	0.0120193	0.6860067
denovo287355	Firmicutes; Clostridia; Clostridiales; Clostridiaceae; Clostridium; NA	OTU	0.5	0.07	0.26 (0.14, 0.49)	2.73E-05	0.0212064	0.76 (0.36, 1.61)	0.4699403	0.9974069	1.24 (0.58, 2.65)	0.5829021	0.9816193
denovo670267	Firmicutes; Clostridia; Clostridiales; Lachnospiraceae; Coprococcus; NA	OTU	2.91	0.03	0.64 (0.5, 0.81)	0.0002113	0.0529539	0.93 (0.64, 1.34)	0.6899157	0.9974069	0.57 (0.38, 0.84)	0.0046425	0.6373929
denovo785907	Firmicutes; Clostridia; Clostridiales; Lachnospiraceae; Coprococcus; NA	OTU	1.24	0.03	0.58 (0.44, 0.78)	0.0002038	0.0529539	0.94 (0.61, 1.44)	0.777574	0.9974069	0.95 (0.62, 1.47)	0.8187952	0.9946789
denovo189398	Firmicutes; Clostridia; Clostridiales; Lachnospiraceae; Dorea; NA	OTU	12.01	6.48	2.54 (1.6, 4.04)	8.30E-05	0.0455492	1.06 (0.55, 2.02)	0.8654718	0.9974069	1.95 (1, 3.81)	0.0494073	0.8164556
denovo166416	Firmicutes; Clostridia; Clostridiales; Lachnospiraceae; NA; NA	OTU	0.27	0.03	0.42 (0.25, 0.71)	0.0009851	0.0921308	0.74 (0.38, 1.46)	0.3888154	0.9974069	0.53 (0.27, 1.06)	0.0741795	0.8744457
denovo333913	Firmicutes; Clostridia; Clostridiales; Lachnospiraceae; NA; NA	OTU	1.97	0.05	0.52 (0.37, 0.74)	0.0002494	0.0529539	0.79 (0.47, 1.33)	0.3812259	0.9974069	1.03 (0.61, 1.74)	0.9218755	0.9969289
denovo442772	Firmicutes; Clostridia; Clostridiales; Lachnospiraceae; NA; NA	OTU	8.35	0.06	2.03 (1.39, 2.97)	0.0002673	0.0529539	1.71 (0.98, 2.99)	0.0598828	0.9974069	1.4 (0.78, 2.52)	0.2594323	0.9667013
denovo501253	Firmicutes; Clostridia; Clostridiales; Lachnospiraceae; NA; NA	OTU	0.67	0.02	0.54 (0.38, 0.77)	0.0005793	0.0652509	0.85 (0.51, 1.42)	0.5348151	0.9974069	0.84 (0.51, 1.4)	0.5124426	0.9816193
denovo521268	Firmicutes; Clostridia; Clostridiales; Lachnospiraceae; NA; NA	OTU	1.57	1.9	0.23 (0.12, 0.43)	5.06E-06	0.0059039	0.52 (0.24, 1.11)	0.0885387	0.9974069	0.3 (0.14, 0.65)	0.0023442	0.6028575
denovo74320	Firmicutes; Clostridia; Clostridiales; Lachnospiraceae; NA; NA	OTU	5.64	0.47	0.62 (0.47, 0.81)	0.0005871	0.0652509	0.32 (0.21, 0.51)	8.00E-07	0.0018611	0.67 (0.43, 1.05)	0.0840296	0.8846448
denovo930802	Firmicutes; Clostridia; Clostridiales; Lachnospiraceae; NA; NA	OTU	0.32	0.04	0.37 (0.22, 0.61)	9.76E-05	0.0455492	0.94 (0.49, 1.81)	0.8632145	0.9974069	1.34 (0.7, 2.54)	0.3745806	0.9816193
denovo507939	Firmicutes; Clostridia; Clostridiales; NA; NA; NA	OTU	1.26	0.08	0.37 (0.2, 0.67)	0.0009868	0.0921308	0.56 (0.27, 1.18)	0.1269506	0.9974069	0.77 (0.36, 1.63)	0.4961934	0.9816193
denovo556761	Firmicutes; Clostridia; Clostridiales; NA; NA; NA	OTU	1.33	0.07	0.58 (0.43, 0.79)	0.0004538	0.0588485	1.39 (0.89, 2.17)	0.1486189	0.9974069	0.82 (0.51, 1.32)	0.4140237	0.9816193
denovo661611	Firmicutes; Clostridia; Clostridiales; NA; NA; NA	OTU	8.24	0.14	0.34 (0.19, 0.61)	0.0003075	0.0529539	0.43 (0.2, 0.91)	0.0269111	0.9974069	0.52 (0.25, 1.11)	0.0913407	0.8995322
denovo787660	Firmicutes; Clostridia; Clostridiales; NA; NA; NA	OTU	2.36	0.01	0.34 (0.19, 0.6)	0.0001547	0.0529539	0.57 (0.28, 1.17)	0.1255377	0.9974069	0.88 (0.42, 1.84)	0.7407964	0.9944157
denovo918201	Firmicutes; Clostridia; Clostridiales; NA; NA; NA	OTU	1.08	0.83	0.33 (0.18, 0.6)	0.0003347	0.0529539	0.64 (0.3, 1.36)	0.2433705	0.9974069	0.51 (0.24, 1.1)	0.0861417	0.885704
denovo12383	Firmicutes; Clostridia; Clostridiales; Ruminococcaceae; NA; NA	OTU	2.16	0.02	0.15 (0.08, 0.27)	1.68E-09	3.92E-06	0.33 (0.16, 0.71)	0.0041741	0.7609434	0.71 (0.33, 1.51)	0.3722412	0.9816193
denovo294260	Firmicutes; Clostridia; Clostridiales; Ruminococcaceae; NA; NA	OTU	2.69	0.08	0.52 (0.36, 0.74)	0.0003461	0.0529539	0.88 (0.52, 1.49)	0.6238931	0.9974069	1.63 (0.94, 2.8)	0.0795624	0.8744457
denovo327852	Firmicutes; Clostridia; Clostridiales; Ruminococcaceae; NA; NA	OTU	1.44	0.39	0.42 (0.25, 0.7)	0.0007856	0.0797238	0.9 (0.46, 1.76)	0.7520327	0.9974069	0.84 (0.42, 1.67)	0.6171457	0.9816193
denovo381284	Firmicutes; Clostridia; Clostridiales; Ruminococcaceae; NA; NA	OTU	0.83	0.08	0.49 (0.34, 0.71)	0.0001804	0.0529539	0.8 (0.47, 1.36)	0.4104935	0.9974069	1.57 (0.93, 2.65)	0.0936223	0.9080103
denovo699246	Firmicutes; Clostridia; Clostridiales; Ruminococcaceae; NA; NA	OTU	2.06	0.52	0.42 (0.26, 0.67)	0.0003301	0.0529539	0.61 (0.32, 1.18)	0.1434552	0.9974069	1.48 (0.76, 2.89)	0.2451824	0.9667013

Supplementary Table 5. Differentially abundant taxa (phylum-genus levels) between controls and conventional adenoma cases, hyperplastic polyp cases, or SSA cases, as detected by the DESeq function in the DESeq2 package. Models were adjusted for age, sex, categorical BMI, and study. All taxa with an FDR-adjusted q<0.10 are included in the table.

					**Conventional adenoma cases vs. controls**			**Hyperplastic polyp cases vs. controls**			**SSA cases vs. controls**		
**Taxon name**	**Taxon classification**	**Level**	**Mean normalized count**	**Maximum Cook's distance**	**Fold change (95% CI)**	**p-value**	**q-value**	**Fold change (95% CI)**	**p-value**	**q-value**	**Fold change (95% CI)**	**p-value**	**q-value**
denovo783221	Actinobacteria; Actinobacteria; Actinomycetales	Order	6.44	1.18	1.87 (1.45, 2.4)	1.00E-06	1.05E-05	1.29 (0.85, 1.96)	0.2280748	0.9252491	0.98 (0.63, 1.53)	0.9445333	0.9445333
denovo783221	Actinobacteria; Actinobacteria; Actinomycetales; Actinomycetaceae	Family	6.99	0.23	1.51 (1.18, 1.93)	0.0010002	0.008185	1.06 (0.7, 1.58)	0.7949538	0.9909394	1 (0.65, 1.53)	0.9983433	0.9983433
denovo783221	Actinobacteria; Actinobacteria; Actinomycetales; Actinomycetaceae; Actinomyces	Genus	9.09	0.58	1.69 (1.32, 2.17)	3.19E-05	0.0006215	1.24 (0.82, 1.87)	0.2985497	0.9899234	1.07 (0.7, 1.64)	0.7524244	0.9764617
denovo184823	Actinobacteria; Actinobacteria; Actinomycetales; Corynebacteriaceae	Family	0.93	1.35	3.3 (1.67, 6.51)	0.0005737	0.0061678	0.75 (0.27, 2.09)	0.5880726	0.9909394	0.91 (0.32, 2.57)	0.8605133	0.9923738
denovo184823	Actinobacteria; Actinobacteria; Actinomycetales; Corynebacteriaceae; Corynebacterium	Genus	1.03	2	3.73 (1.88, 7.4)	0.0001705	0.0022162	0.78 (0.28, 2.22)	0.6465213	0.9899234	0.86 (0.3, 2.5)	0.7873682	0.9764617
denovo199602	Cyanobacteria	Phylum	44.75	0.01	0.46 (0.26, 0.81)	0.0076452	0.0764519	0.59 (0.26, 1.37)	0.2224569	0.5630235	0.81 (0.33, 1.96)	0.6378759	0.8554366
denovo317079	Firmicutes; Bacilli	Class	506.27	2.31	2.11 (1.6, 2.78)	1.40E-07	2.45E-06	1.23 (0.78, 1.94)	0.3707783	0.9961489	0.79 (0.49, 1.3)	0.3582352	0.7884667
denovo317079	Firmicutes; Bacilli; Lactobacillales	Order	468.25	2.31	2.25 (1.68, 3.02)	7.00E-08	1.51E-06	1.31 (0.81, 2.12)	0.2699278	0.9252491	0.73 (0.43, 1.22)	0.2319356	0.8684908
denovo317079	Firmicutes; Bacilli; Lactobacillales; Streptococcaceae	Family	377.94	1.04	2.03 (1.52, 2.73)	2.05E-06	4.40E-05	1.09 (0.68, 1.75)	0.7077718	0.9909394	0.66 (0.4, 1.1)	0.1136901	0.5431862
denovo317079	Firmicutes; Bacilli; Lactobacillales; Streptococcaceae; Streptococcus	Genus	422.22	2.79	2.38 (1.76, 3.21)	2.00E-08	1.35E-06	1.26 (0.78, 2.05)	0.3450591	0.9899234	0.72 (0.43, 1.21)	0.2163889	0.6491668
denovo220332	Firmicutes; Clostridia; Clostridiales; [Mogibacteriaceae]	Family	17.61	0.91	0.8 (0.66, 0.96)	0.015749	0.0752451	0.8 (0.59, 1.08)	0.142205	0.679424	0.89 (0.64, 1.23)	0.4732812	0.8640164
denovo220332	Firmicutes; Clostridia; Clostridiales; [Mogibacteriaceae]; NA	Genus	16.24	0.15	0.78 (0.65, 0.94)	0.0079306	0.0618584	0.85 (0.63, 1.15)	0.2987712	0.9899234	0.89 (0.65, 1.22)	0.4524979	0.8823709
denovo784890	Firmicutes; Clostridia; Clostridiales; [Tissierellaceae]	Family	6.72	7.33	2.87 (1.7, 4.83)	7.63E-05	0.0010929	0.53 (0.23, 1.2)	0.1264144	0.679424	0.9 (0.39, 2.09)	0.8042368	0.9923738
denovo113729	Firmicutes; Clostridia; Clostridiales; [Tissierellaceae]; Peptoniphilus	Genus	1.6	3.92	3.41 (1.83, 6.35)	0.0001158	0.001807	0.55 (0.21, 1.46)	0.2297883	0.9899234	0.85 (0.32, 2.28)	0.7482896	0.9764617
denovo871288	Firmicutes; Clostridia; Clostridiales; Lachnospiraceae; Anaerostipes	Genus	45.7	0.51	1.23 (0.98, 1.54)	0.0801276	0.297617	1.96 (1.36, 2.84)	0.0003465	0.0270255	1.02 (0.69, 1.52)	0.9201848	0.9795153
denovo23546	Firmicutes; Clostridia; Clostridiales; Lachnospiraceae; Dorea	Genus	216.11	0.09	1.19 (1.05, 1.35)	0.0073688	0.0618584	1.09 (0.88, 1.34)	0.4269694	0.9899234	1 (0.8, 1.25)	0.980611	0.9933462
denovo171687	Firmicutes; Clostridia; Clostridiales; NA	Family	2415.19	1.89	1.23 (1.08, 1.39)	0.0011421	0.008185	1.1 (0.9, 1.35)	0.3630524	0.9695146	0.99 (0.8, 1.23)	0.926627	0.9923738
denovo171687	Firmicutes; Clostridia; Clostridiales; NA; NA	Genus	2624.01	3.99	1.44 (1.26, 1.65)	1.60E-07	6.31E-06	1.18 (0.94, 1.47)	0.1566656	0.9182145	0.98 (0.77, 1.25)	0.8875646	0.9795153
denovo422375	Firmicutes; Clostridia; Clostridiales; Veillonellaceae	Family	482.77	7.34	1.86 (1.44, 2.39)	1.64E-06	4.40E-05	0.79 (0.52, 1.18)	0.2493526	0.8935133	0.66 (0.43, 1.03)	0.067656	0.513957
denovo437434	Firmicutes; Clostridia; Clostridiales; Veillonellaceae; Phascolarctobacterium	Genus	115.38	5.19	1.91 (1.24, 2.96)	0.0035811	0.0399038	1.12 (0.57, 2.22)	0.7434821	0.9899234	0.85 (0.41, 1.76)	0.6574771	0.9764617
denovo628837	Firmicutes; Erysipelotrichi	Class	305.79	0.01	0.92 (0.79, 1.07)	0.2658247	0.5561808	1.1 (0.85, 1.41)	0.468776	0.9961489	0.68 (0.52, 0.89)	0.0053337	0.0906733
denovo973076	Firmicutes; Erysipelotrichi; Erysipelotrichales; Erysipelotrichaceae; Coprobacillus	Genus	10.77	0.12	0.59 (0.39, 0.88)	0.0093364	0.0662034	0.36 (0.19, 0.68)	0.0017059	0.0665298	1.03 (0.53, 2.01)	0.927493	0.9795153
denovo880054	Proteobacteria; Betaproteobacteria; Burkholderiales; Alcaligenaceae	Family	312.49	4.09	1.5 (1.12, 2)	0.0060492	0.0371593	0.83 (0.52, 1.32)	0.4240249	0.9909394	0.82 (0.5, 1.36)	0.4478915	0.8640164
denovo880054	Proteobacteria; Betaproteobacteria; Burkholderiales; Alcaligenaceae; Sutterella	Genus	430.21	8	2.06 (1.52, 2.78)	3.31E-06	8.60E-05	0.84 (0.52, 1.37)	0.4860878	0.9899234	0.97 (0.58, 1.65)	0.922579	0.9795153
denovo846374	Proteobacteria; Gammaproteobacteria	Class	394.44	2.99	1.81 (1.19, 2.76)	0.0052077	0.044265	0.32 (0.16, 0.63)	0.001045	0.0177653	0.43 (0.21, 0.88)	0.0217273	0.1846822
denovo846374	Proteobacteria; Gammaproteobacteria; Enterobacteriales	Order	364.65	8.69	2.05 (1.23, 3.4)	0.005634	0.0317585	0.22 (0.1, 0.49)	0.0002499	0.0052486	0.45 (0.19, 1.08)	0.0735366	0.7498433
denovo789089	Tenericutes; Mollicutes; RF39	Order	40.28	0.03	0.4 (0.21, 0.77)	0.0060492	0.0317585	1.08 (0.39, 2.98)	0.8806008	0.9991107	1.47 (0.5, 4.33)	0.4876755	0.8684908
denovo789089	Tenericutes; Mollicutes; RF39; NA	Family	35.61	0.05	0.43 (0.23, 0.8)	0.0078894	0.0424057	1.06 (0.42, 2.65)	0.9025852	0.9909394	1.12 (0.43, 2.94)	0.8177589	0.9923738
denovo789089	Tenericutes; Mollicutes; RF39; NA; NA	Genus	36.41	0.06	0.42 (0.22, 0.78)	0.0061691	0.0601487	1.1 (0.44, 2.77)	0.8425117	0.9899234	1.06 (0.4, 2.81)	0.909297	0.9795153

Supplementary Table 6. Differentially abundant taxa (phylum-OTU level) between controls and proximal or distal conventional adenoma cases, as detected by the DESeq function in the DESeq2 package. Models were adjusted for age, sex, categorical BMI, and study. All taxa with an FDR-adjusted q<0.10 are included in the table.

					**Proximal conventional adenoma cases vs. controls**			**Distal conventional adenoma cases vs. controls**		
**Taxon name**	**Taxon classification**	**Level**	**Mean normalized count**	**Maximum Cook's Distance**	**Fold change (95% CI)**	**p-value**	**q-value**	**Fold change (95% CI)**	**p-value**	**q-value**
denovo783221	Actinobacteria; Actinobacteria; Actinomycetales	Order	6.46	0.99	1.52 (1.32, 1.75)	9.22E-09	9.68E-08	1.08 (0.91, 1.3)	0.3747515	0.6938279
denovo783221	Actinobacteria; Actinobacteria; Actinomycetales; Actinomycetaceae	Family	7.01	0.26	1.27 (1.1, 1.47)	0.0015186	0.0095455	1.19 (1, 1.42)	0.0534649	0.1960381
denovo783221	Actinobacteria; Actinobacteria; Actinomycetales; Actinomycetaceae; Actinomyces	Genus	9.12	0.54	1.39 (1.2, 1.61)	1.18E-05	0.0001281	1.18 (0.99, 1.42)	0.0638526	0.2855846
denovo184823	Actinobacteria; Actinobacteria; Actinomycetales; Corynebacteriaceae	Family	0.94	1.05	2.57 (1.74, 3.78)	1.70E-06	1.87E-05	0.8 (0.5, 1.28)	0.3503681	0.7029844
denovo184823	Actinobacteria; Actinobacteria; Actinomycetales; Corynebacteriaceae; Corynebacterium	Genus	1.03	1.64	2.81 (1.9, 4.16)	2.40E-07	4.56E-06	0.83 (0.51, 1.34)	0.4389869	0.7330248
denovo653463	Bacteroidetes; Bacteroidia; Bacteroidales; [Odoribacteraceae]	Family	87.46	3.44	1.28 (1.06, 1.56)	0.0123021	0.0601437	0.92 (0.73, 1.16)	0.496499	0.840229
denovo653463	Bacteroidetes; Bacteroidia; Bacteroidales; [Odoribacteraceae]; Odoribacter	Genus	58.06	3.82	1.43 (1.15, 1.79)	0.0015733	0.0119574	1 (0.77, 1.31)	0.9803745	0.9997171
denovo2101	Bacteroidetes; Bacteroidia; Bacteroidales; Bacteroidaceae; Bacteroides; NA	OTU	0.41	0.17	0.59 (0.44, 0.77)	0.0001709	0.0613667	1.09 (0.75, 1.59)	0.6514445	0.9098944
denovo724396	Bacteroidetes; Bacteroidia; Bacteroidales; Porphyromonadaceae; Porphyromonas	Genus	0.69	1.08	2 (1.21, 3.3)	0.0067157	0.037648	0.77 (0.44, 1.35)	0.3648339	0.7330248
denovo317079	Firmicutes; Bacilli	Class	507.66	3.01	1.67 (1.42, 1.95)	2.51E-10	4.26E-09	1.08 (0.89, 1.32)	0.4371713	0.7323682
denovo317079	Firmicutes; Bacilli; Lactobacillales	Order	469.58	2.92	1.74 (1.47, 2.06)	9.50E-11	1.99E-09	1.09 (0.88, 1.34)	0.4295125	0.6938279
denovo665441	Firmicutes; Bacilli; Lactobacillales; Enterococcaceae	Family	4.8	3.54	1.12 (0.8, 1.57)	0.5204717	0.6735517	0.49 (0.33, 0.74)	0.0006917	0.0152164
denovo665441	Firmicutes; Bacilli; Lactobacillales; Enterococcaceae; Enterococcus	Genus	6.11	2.73	1.1 (0.78, 1.56)	0.580253	0.8817962	0.48 (0.32, 0.73)	0.0005673	0.0143718
denovo317079	Firmicutes; Bacilli; Lactobacillales; Streptococcaceae	Family	379.01	1.25	1.58 (1.32, 1.88)	3.67E-07	5.38E-06	1.24 (1.01, 1.53)	0.0398003	0.1731513
denovo317079	Firmicutes; Bacilli; Lactobacillales; Streptococcaceae; Streptococcus	Genus	423.53	6.55	1.84 (1.54, 2.2)	3.05E-11	1.16E-09	1.21 (0.98, 1.5)	0.076235	0.2896929
denovo945918	Firmicutes; Bacilli; Lactobacillales; Streptococcaceae; Streptococcus; NA	OTU	4.87	0.19	1.19 (1, 1.43)	0.0556381	0.6611284	1.55 (1.23, 1.94)	0.0001729	0.0309794
denovo210003	Firmicutes; Clostridia	Class	16497.05	0.03	0.99 (0.95, 1.03)	0.5200031	0.7712438	0.94 (0.89, 0.98)	0.0094297	0.0801527
denovo220332	Firmicutes; Clostridia; Clostridiales; [Mogibacteriaceae]	Family	17.62	0.82	0.95 (0.85, 1.06)	0.3296888	0.5949894	0.81 (0.71, 0.92)	0.0018629	0.0273219
denovo220332	Firmicutes; Clostridia; Clostridiales; [Mogibacteriaceae]; NA	Genus	16.25	0.13	0.93 (0.83, 1.04)	0.1913103	0.4846526	0.81 (0.71, 0.93)	0.0022721	0.0373499
denovo380858	Firmicutes; Clostridia; Clostridiales; [Mogibacteriaceae]; NA; NA	OTU	0.76	0.07	0.96 (0.78, 1.18)	0.7066747	0.9814869	0.58 (0.43, 0.78)	0.0003031	0.0441196
denovo784890	Firmicutes; Clostridia; Clostridiales; [Tissierellaceae]	Family	6.74	4.46	2.26 (1.66, 3.07)	2.08E-07	4.58E-06	1.01 (0.7, 1.45)	0.9663512	0.9663512
denovo712300	Firmicutes; Clostridia; Clostridiales; [Tissierellaceae]; Anaerococcus	Genus	2.03	7.03	1.96 (1.26, 3.07)	0.0030127	0.0208151	1 (0.6, 1.65)	0.9997171	0.9997171
denovo113729	Firmicutes; Clostridia; Clostridiales; [Tissierellaceae]; Peptoniphilus	Genus	1.61	2.19	2.56 (1.78, 3.67)	3.81E-07	5.80E-06	1.02 (0.66, 1.56)	0.9398665	0.9784912
denovo113729	Firmicutes; Clostridia; Clostridiales; [Tissierellaceae]; Peptoniphilus; NA	OTU	0.72	2.2	1.71 (1.3, 2.25)	0.0001189	0.0613667	0.97 (0.65, 1.44)	0.8687172	0.9702102
denovo903712	Firmicutes; Clostridia; Clostridiales; Christensenellaceae	Family	102.75	0.29	1.14 (0.94, 1.39)	0.1933383	0.4477307	0.58 (0.46, 0.73)	5.94E-06	0.0002613
denovo903712	Firmicutes; Clostridia; Clostridiales; Christensenellaceae; NA	Genus	104.15	0.32	1.22 (1, 1.49)	0.0531495	0.2019681	0.56 (0.44, 0.71)	2.13E-06	0.000162
denovo643232	Firmicutes; Clostridia; Clostridiales; Christensenellaceae; NA; NA	OTU	0.34	0.24	0.95 (0.71, 1.25)	0.695777	0.9814869	0.47 (0.31, 0.72)	0.0005313	0.058919
denovo953537	Firmicutes; Clostridia; Clostridiales; Christensenellaceae; NA; NA	OTU	6.42	0.08	1.11 (0.83, 1.49)	0.4694827	0.9750606	0.41 (0.27, 0.64)	7.43E-05	0.0234869
denovo56002	Firmicutes; Clostridia; Clostridiales; Clostridiaceae	Family	259.61	0.07	1.11 (0.96, 1.28)	0.1762487	0.4477307	0.79 (0.66, 0.94)	0.0071677	0.0788449
denovo140819	Firmicutes; Clostridia; Clostridiales; Clostridiaceae; Clostridium	Genus	72.45	0.22	1.41 (1.17, 1.71)	0.0002983	0.0028336	0.64 (0.51, 0.8)	9.37E-05	0.0035595
denovo140819	Firmicutes; Clostridia; Clostridiales; Clostridiaceae; Clostridium; NA	OTU	33.98	0.03	0.94 (0.72, 1.23)	0.6491011	0.9814869	0.48 (0.33, 0.69)	9.40E-05	0.0243261
denovo287355	Firmicutes; Clostridia; Clostridiales; Clostridiaceae; Clostridium; NA	OTU	0.51	0.07	0.67 (0.51, 0.89)	0.0056083	0.344409	0.47 (0.31, 0.71)	0.0003772	0.0516814
denovo871288	Firmicutes; Clostridia; Clostridiales; Lachnospiraceae; Anaerostipes	Genus	45.76	0.15	1.21 (1.05, 1.38)	0.0069351	0.037648	0.94 (0.8, 1.11)	0.4822532	0.7330248
denovo670267	Firmicutes; Clostridia; Clostridiales; Lachnospiraceae; Coprococcus;	OTU	2.91	0.03	0.77 (0.67, 0.89)	0.0002325	0.0676854	0.85 (0.72, 1)	0.0559323	0.4710579
denovo636353	Firmicutes; Clostridia; Clostridiales; Lachnospiraceae; Coprococcus; NA	OTU	0.41	0.08	0.65 (0.52, 0.81)	0.0001164	0.0613667	0.92 (0.71, 1.18)	0.5020466	0.8809658
denovo23546	Firmicutes; Clostridia; Clostridiales; Lachnospiraceae; Dorea	Genus	216.34	0.08	1.13 (1.05, 1.22)	0.0015602	0.0119574	1.03 (0.94, 1.13)	0.5060294	0.754083
denovo189398	Firmicutes; Clostridia; Clostridiales; Lachnospiraceae; Dorea; NA	OTU	12.05	8.25	1.65 (1.3, 2.09)	3.18E-05	0.0370198	1.23 (0.9, 1.68)	0.1941341	0.6890648
denovo271591	Firmicutes; Clostridia; Clostridiales; Lachnospiraceae; NA; NA	OTU	0.59	0.01	0.64 (0.5, 0.82)	0.0002965	0.0720472	0.83 (0.61, 1.13)	0.240991	0.739742
denovo442772	Firmicutes; Clostridia; Clostridiales; Lachnospiraceae; NA; NA	OTU	8.33	0.05	1.25 (1.02, 1.53)	0.0345755	0.6048153	1.58 (1.21, 2.05)	0.000657	0.0665318
denovo871995	Firmicutes; Clostridia; Clostridiales; Lachnospiraceae; NA; NA	OTU	0.22	0.03	0.61 (0.48, 0.79)	0.0001669	0.0613667	0.95 (0.7, 1.29)	0.724955	0.9400822
denovo171687	Firmicutes; Clostridia; Clostridiales; NA	Family	2413.95	1.84	1.18 (1.1, 1.27)	1.10E-05	9.69E-05	0.99 (0.91, 1.08)	0.8450368	0.9533748
denovo171687	Firmicutes; Clostridia; Clostridiales; NA; NA	Genus	2624.72	3.55	1.34 (1.23, 1.45)	1.30E-12	9.95E-11	0.99 (0.9, 1.09)	0.8347717	0.9194587
denovo142312	Firmicutes; Clostridia; Clostridiales; NA; NA; NA	OTU	0.62	0.09	0.84 (0.64, 1.1)	0.1996481	0.8438458	0.44 (0.29, 0.65)	4.69E-05	0.0218579
denovo610921	Firmicutes; Clostridia; Clostridiales; NA; NA; NA	OTU	0.42	0	0.9 (0.68, 1.21)	0.4946244	0.9750606	0.45 (0.29, 0.71)	0.0004426	0.0542566
denovo661611	Firmicutes; Clostridia; Clostridiales; NA; NA; NA	OTU	8.27	0.08	0.53 (0.4, 0.7)	5.15E-06	0.0120019	0.92 (0.63, 1.35)	0.6646136	0.9148257
denovo753166	Firmicutes; Clostridia; Clostridiales; NA; NA; NA	OTU	2.75	0.02	0.92 (0.7, 1.22)	0.5755434	0.9785333	0.49 (0.33, 0.74)	0.000572	0.0605495
denovo231391	Firmicutes; Clostridia; Clostridiales; Ruminococcaceae; Faecalibacterium; prausnitzii	OTU	0.67	0.06	0.92 (0.72, 1.18)	0.5213519	0.9785333	0.46 (0.32, 0.67)	4.13E-05	0.0218579
denovo1016938	Firmicutes; Clostridia; Clostridiales; Ruminococcaceae; NA; NA	OTU	0.51	0.02	0.91 (0.71, 1.18)	0.4816221	0.9750606	0.5 (0.34, 0.72)	0.0002834	0.0440046
denovo12383	Firmicutes; Clostridia; Clostridiales; Ruminococcaceae; NA; NA	OTU	2.17	0.02	0.59 (0.44, 0.78)	0.0001844	0.0613667	0.31 (0.2, 0.47)	2.77E-08	6.44E-05
denovo294260	Firmicutes; Clostridia; Clostridiales; Ruminococcaceae; NA; NA	OTU	2.7	0.06	0.82 (0.68, 1)	0.0491015	0.6598671	0.57 (0.43, 0.74)	2.10E-05	0.0162921
denovo327852	Firmicutes; Clostridia; Clostridiales; Ruminococcaceae; NA; NA	OTU	1.45	0.4	0.77 (0.6, 0.99)	0.0399381	0.6131719	0.53 (0.37, 0.76)	0.0004979	0.0579793
denovo381284	Firmicutes; Clostridia; Clostridiales; Ruminococcaceae; NA; NA	OTU	0.83	0.07	0.69 (0.56, 0.84)	0.0003093	0.0720472	0.78 (0.61, 1.01)	0.0590165	0.4732228

denovo411531	Firmicutes; Clostridia; Clostridiales; Ruminococcaceae; NA; NA	OTU	0.72	0.06	0.86 (0.66, 1.13)	0.2786098	0.9126333	0.45 (0.3, 0.67)	7.53E-05	0.0234869
denovo699246	Firmicutes; Clostridia; Clostridiales; Ruminococcaceae; NA; NA	OTU	2.07	0.54	0.8 (0.63, 1.01)	0.0649682	0.7037715	0.48 (0.34, 0.67)	1.85E-05	0.0162921
denovo705914	Firmicutes; Clostridia; Clostridiales; Ruminococcaceae; NA; NA	OTU	13.08	0.04	0.91 (0.71, 1.18)	0.4718418	0.9750606	0.49 (0.35, 0.7)	8.07E-05	0.0234869
denovo879175	Firmicutes; Clostridia; Clostridiales; Ruminococcaceae; NA; NA	OTU	0.73	0.04	0.9 (0.72, 1.13)	0.3822873	0.9491974	0.57 (0.41, 0.79)	0.0007025	0.0681703
denovo93364	Firmicutes; Clostridia; Clostridiales; Ruminococcaceae; NA; NA	OTU	0.6	0.16	0.97 (0.73, 1.27)	0.8097527	0.9878961	0.45 (0.3, 0.68)	0.0001306	0.0287557
denovo326882	Firmicutes; Clostridia; Clostridiales; Ruminococcaceae; Oscillospira; NA	OTU	0.66	0.02	0.88 (0.69, 1.12)	0.3138993	0.9287089	0.55 (0.39, 0.78)	0.0008283	0.0771687
denovo228423	Firmicutes; Clostridia; Clostridiales; Ruminococcaceae; Ruminococcus; NA	OTU	0.62	0.01	0.98 (0.76, 1.28)	0.9024729	0.988145	0.48 (0.33, 0.7)	0.0001489	0.028907
denovo326096	Firmicutes; Clostridia; Clostridiales; Ruminococcaceae; Ruminococcus; NA	OTU	36.13	0.14	1.04 (0.78, 1.38)	0.7932217	0.9859538	0.44 (0.29, 0.67)	0.0001358	0.0287557
denovo859546	Firmicutes; Clostridia; Clostridiales; Ruminococcaceae; Ruminococcus; NA	OTU	1.04	0.01	1.06 (0.82, 1.37)	0.654051	0.9814869	0.52 (0.36, 0.75)	0.0004399	0.0542566
denovo998823	Firmicutes; Clostridia; Clostridiales; Ruminococcaceae; Ruminococcus; NA	OTU	0.55	0.03	1.08 (0.82, 1.42)	0.5883983	0.9801843	0.46 (0.3, 0.7)	0.0002816	0.0440046
denovo422375	Firmicutes; Clostridia; Clostridiales; Veillonellaceae	Family	484.27	6.04	1.72 (1.48, 2)	1.00E-12	4.51E-11	0.99 (0.83, 1.18)	0.8910024	0.9663512
denovo437434	Firmicutes; Clostridia; Clostridiales; Veillonellaceae; Phascolarctobacterium	Genus	115.54	4.75	1.83 (1.41, 2.37)	6.03E-06	7.63E-05	0.96 (0.71, 1.3)	0.7980424	0.9168133
denovo50244	Firmicutes; Erysipelotrichi; Erysipelotrichales; Erysipelotrichaceae; cc_115	Genus	5.07	0.1	0.91 (0.64, 1.29)	0.6033342	0.8817962	0.53 (0.35, 0.8)	0.0024572	0.0373499
denovo973076	Firmicutes; Erysipelotrichi; Erysipelotrichales; Erysipelotrichaceae; Coprobacillus	Genus	10.81	0.11	0.71 (0.56, 0.9)	0.0051427	0.0325703	0.88 (0.66, 1.16)	0.3553808	0.7330248
denovo188989	Proteobacteria; Alphaproteobacteria; RF32; NA	Family	117.05	0.01	0.6 (0.42, 0.86)	0.0050957	0.0280265	0.91 (0.6, 1.37)	0.6490405	0.8653874
denovo188989	Proteobacteria; Alphaproteobacteria; RF32; NA; NA	Genus	115.36	0.01	0.63 (0.44, 0.9)	0.0124022	0.062838	0.92 (0.61, 1.4)	0.7041616	0.8494647
denovo880054	Proteobacteria; Betaproteobacteria; Burkholderiales; Alcaligenaceae	Family	313.06	3.67	1.44 (1.21, 1.71)	3.01E-05	0.0002207	1 (0.81, 1.22)	0.9656724	0.9663512
denovo880054	Proteobacteria; Betaproteobacteria; Burkholderiales; Alcaligenaceae; Sutterella	Genus	431.23	7.04	1.81 (1.51, 2.17)	1.14E-10	2.88E-09	1.11 (0.9, 1.37)	0.342848	0.7330248
denovo846374	Proteobacteria; Gammaproteobacteria	Class	395.71	2.49	1.55 (1.24, 1.94)	0.0001459	0.0012404	0.94 (0.7, 1.26)	0.6945111	0.8850375
denovo846374	Proteobacteria; Gammaproteobacteria; Enterobacteriales	Order	365.82	7.84	1.6 (1.23, 2.09)	0.000573	0.0040111	1.02 (0.71, 1.45)	0.9217112	0.953799
denovo948713	Synergistetes	Phylum	2.53	7.17	1.08 (0.92, 1.25)	0.3488038	0.7161231	0.49 (0.29, 0.84)	0.0093674	0.0936743
denovo654064	Tenericutes; Mollicutes	Class	75.66	0.75	1.38 (1.02, 1.88)	0.0398944	0.1695511	0.54 (0.35, 0.84)	0.0065296	0.0801527

Supplementary Table 7. Differentially abundant taxa (phylum-OTU level) between controls and non-advanced or advanced conventional adenoma cases, as detected by the DESeq function in the DESeq2 package. Models were adjusted for age, sex, categorical BMI, and study. All taxa with an FDR-adjusted q<0.10 are included in the table.

					Non-advanced conventional adenoma cases vs. controls			Advanced conventional adenoma cases vs. controls		
Taxon name	Taxon classification	Level	Mean normalized count	Maximum Cook's Distance	Fold change (95% CI)	p-value	q-value	Fold change (95% CI)	p-value	q-value
denovo783221	Actinobacteria; Actinobacteria; Actinomycetales	Order	6.42	0.69	1.28 (0.99, 1.65)	0.0648277	0.2715552	5.38 (3.24, 8.92)	7.00E-11	1.57E-09
denovo783221	Actinobacteria; Actinobacteria; Actinomycetales; Actinomycetaceae	Family	6.95	0.25	1.36 (1.05, 1.77)	0.0204213	0.179707	2.22 (1.31, 3.78)	0.0031626	0.0278311
denovo783221	Actinobacteria; Actinobacteria; Actinomycetales; Actinomycetaceae; Actinomyces	Genus	9.08	0.65	1.57 (1.2, 2.04)	0.0008837	0.0139617	2.35 (1.38, 4.01)	0.0017422	0.019662
denovo184823	Actinobacteria; Actinobacteria; Actinomycetales; Corynebacteriaceae	Family	0.94	1.1	1.17 (0.57, 2.39)	0.6690566	0.8920754	15.32 (4.53, 51.78)	1.12E-05	0.0002454
denovo184823	Actinobacteria; Actinobacteria; Actinomycetales; Corynebacteriaceae; Corynebacterium	Genus	1.03	1.68	1.28 (0.62, 2.62)	0.5068122	0.7850621	16.69 (5.04, 55.28)	4.06E-06	0.000107
denovo670950	Bacteroidetes; Bacteroidia; Bacteroidales; [Barnesiellaceae]	Family	117.2	0.06	0.97 (0.59, 1.58)	0.8965212	0.9786016	0.3 (0.12, 0.76)	0.01167	0.0649406
denovo670950	Bacteroidetes; Bacteroidia; Bacteroidales; [Barnesiellaceae]; NA	Genus	117.29	0.04	1.03 (0.63, 1.68)	0.902878	0.9865789	0.31 (0.12, 0.78)	0.0128032	0.0957327
denovo653463	Bacteroidetes; Bacteroidia; Bacteroidales; [Odoribacteraceae]; Odoribacter	Genus	57.73	3.73	1.71 (1.14, 2.54)	0.0087086	0.098283	0.59 (0.27, 1.29)	0.1884404	0.4841002
denovo724396	Bacteroidetes; Bacteroidia; Bacteroidales; Porphyromonadaceae; Porphyromonas	Genus	0.68	1	0.59 (0.24, 1.44)	0.2423762	0.6331259	9.78 (2.42, 39.48)	0.0013662	0.0184341
denovo199602	Cyanobacteria	Phylum	44.83	0.01	0.52 (0.29, 0.95)	0.0343972	0.3439724	0.25 (0.1, 0.65)	0.0046533	0.0465331
denovo317079	Firmicutes; Bacilli	Class	505.44	2.38	2.16 (1.61, 2.9)	3.15E-07	5.35E-06	1.85 (1.03, 3.33)	0.0410949	0.1746532
denovo317079	Firmicutes; Bacilli; Lactobacillales	Order	467.44	2.33	2.29 (1.67, 3.14)	2.23E-07	4.69E-06	2.05 (1.1, 3.82)	0.024549	0.1031057
denovo194049	Firmicutes; Bacilli; Lactobacillales; Lactobacillaceae; Lactobacillus; zeae	OTU	1.92	5.11	3.73 (1.92, 7.26)	0.0001024	0.0597919	1.32 (0.5, 3.48)	0.5723943	0.8602159
denovo317079	Firmicutes; Bacilli; Lactobacillales; Streptococcaceae	Family	376.64	1.21	2.08 (1.52, 2.85)	4.39E-06	9.65E-05	1.85 (0.99, 3.44)	0.0524198	0.1893391
denovo317079	Firmicutes; Bacilli; Lactobacillales; Streptococcaceae; Streptococcus	Genus	422.04	3.6	2.63 (1.91, 3.62)	3.62E-09	2.15E-07	1.48 (0.78, 2.78)	0.2277994	0.4934009
denovo220332	Firmicutes; Clostridia; Clostridiales; [Mogibacteriaceae]; NA	Genus	16.23	0.13	0.81 (0.67, 0.98)	0.0338923	0.2434084	0.61 (0.41, 0.92)	0.0172183	0.0971602
denovo784890	Firmicutes; Clostridia; Clostridiales; [Tissierellaceae]	Family	6.73	3.73	1.14 (0.66, 1.97)	0.6297732	0.8833394	14.97 (5.48, 40.89)	1.31E-07	5.75E-06
denovo712300	Firmicutes; Clostridia; Clostridiales; [Tissierellaceae]; Anaerococcus	Genus	2.02	6.82	0.9 (0.41, 1.99)	0.7968062	0.9537529	8.67 (2.35, 32.03)	0.0011968	0.0184341
denovo784890	Firmicutes; Clostridia; Clostridiales; [Tissierellaceae]; Finegoldia	Genus	2.26	9.96	1.73 (0.81, 3.72)	0.1578763	0.5321187	28.25 (7.89, 101.09)	2.80E-07	2.22E-05
denovo113729	Firmicutes; Clostridia; Clostridiales; [Tissierellaceae]; Peptoniphilus	Genus	1.61	1.72	1.29 (0.68, 2.45)	0.4417771	0.7425615	17.18 (5.62, 52.46)	6.00E-07	2.37E-05
denovo113729	Firmicutes; Clostridia; Clostridiales; [Tissierellaceae]; Peptoniphilus; NA	OTU	0.71	1.4	1.07 (0.55, 2.06)	0.8457738	0.9884577	9.12 (3.56, 23.33)	4.02E-06	0.0094006
denovo903712	Firmicutes; Clostridia; Clostridiales; Christensenellaceae	Family	102.53	0.36	1 (0.7, 1.43)	0.996671	0.996671	0.3 (0.15, 0.61)	0.0009675	0.0141894
denovo903712	Firmicutes; Clostridia; Clostridiales; Christensenellaceae; NA	Genus	103.95	0.4	1.11 (0.77, 1.6)	0.573488	0.8336451	0.31 (0.15, 0.64)	0.0014001	0.0184341
denovo287355	Firmicutes; Clostridia; Clostridiales; Clostridiaceae; Clostridium; NA	OTU	0.5	0.08	0.24 (0.12, 0.49)	9.44E-05	0.0597919	0.29 (0.11, 0.77)	0.0132321	0.4319583
denovo23546	Firmicutes; Clostridia; Clostridiales; Lachnospiraceae; Dorea	Genus	215.62	0.08	1.21 (1.05, 1.38)	0.0062116	0.081786	1.07 (0.81, 1.4)	0.6499173	0.7844788
denovo189398	Firmicutes; Clostridia; Clostridiales; Lachnospiraceae; Dorea; NA	OTU	12.03	5.73	2.68 (1.61, 4.46)	0.0001405	0.0656498	2.19 (0.95, 5.06)	0.0669658	0.5203333
denovo667264	Firmicutes; Clostridia; Clostridiales; Lachnospiraceae; Lachnobacterium	Genus	7.25	0.1	0.87 (0.64, 1.2)	0.4034635	0.7412468	0.43 (0.22, 0.84)	0.0133299	0.0957327
denovo525776	Firmicutes; Clostridia; Clostridiales; Lachnospiraceae; Lachnospira	Genus	317.71	0.05	0.91 (0.73, 1.14)	0.4348417	0.7425615	0.58 (0.37, 0.9)	0.0162025	0.0971602
denovo521268	Firmicutes; Clostridia; Clostridiales; Lachnospiraceae; NA; NA	OTU	1.58	1.66	0.17 (0.08, 0.36)	2.24E-06	0.0026178	0.35 (0.13, 0.94)	0.0378641	0.5008256
denovo171687	Firmicutes; Clostridia; Clostridiales; NA	Family	2417.97	1.72	1.28 (1.12, 1.46)	0.0002384	0.0034961	1 (0.77, 1.3)	0.9904776	0.9904776
denovo171687	Firmicutes; Clostridia; Clostridiales; NA; NA	Genus	2628.04	3.57	1.54 (1.33, 1.78)	5.43E-09	2.15E-07	1.03 (0.77, 1.38)	0.8428564	0.8823091
denovo12383	Firmicutes; Clostridia; Clostridiales; Ruminococcaceae; NA; NA	OTU	2.16	0.01	0.08 (0.04, 0.17)	1.00E-11	3.26E-08	0.35 (0.13, 0.92)	0.0342761	0.4956075
denovo294260	Firmicutes; Clostridia; Clostridiales; Ruminococcaceae; NA; NA	OTU	2.69	0.06	0.48 (0.33, 0.71)	0.0002483	0.0828736	0.71 (0.35, 1.45)	0.3509341	0.7440348
denovo699246	Firmicutes; Clostridia; Clostridiales; Ruminococcaceae; NA; NA	OTU	2.06	0.51	0.37 (0.22, 0.63)	0.000233	0.0828736	0.56 (0.23, 1.33)	0.1890542	0.6389356
denovo422375	Firmicutes; Clostridia; Clostridiales; Veillonellaceae	Family	476.74	6.28	2.15 (1.65, 2.82)	2.16E-08	9.52E-07	0.82 (0.48, 1.4)	0.4589797	0.5609752
denovo437434	Firmicutes; Clostridia; Clostridiales; Veillonellaceae; Phascolarctobacterium	Genus	115.56	4.63	2.31 (1.45, 3.67)	0.0004435	0.0087597	0.79 (0.33, 1.92)	0.6078289	0.7733044
denovo188989	Proteobacteria; Alphaproteobacteria	Class	128.11	0.03	0.63 (0.34, 1.19)	0.1538695	0.5231564	0.18 (0.06, 0.58)	0.0043142	0.0733417
denovo188989	Proteobacteria; Alphaproteobacteria; RF32	Order	125.53	0.03	0.61 (0.31, 1.17)	0.1350111	0.4050334	0.2 (0.06, 0.66)	0.0083271	0.0501856
denovo188989	Proteobacteria; Alphaproteobacteria; RF32; NA	Family	116.83	0.01	0.58 (0.3, 1.12)	0.1061811	0.3839762	0.21 (0.06, 0.67)	0.0089852	0.0649406
denovo188989	Proteobacteria; Alphaproteobacteria; RF32; NA; NA	Genus	115.14	0.01	0.62 (0.32, 1.19)	0.1485187	0.5321187	0.23 (0.07, 0.73)	0.0125152	0.0957327
denovo880054	Proteobacteria; Betaproteobacteria; Burkholderiales; Alcaligenaceae	Family	312.77	3.63	1.66 (1.22, 2.27)	0.0012249	0.0134734	0.95 (0.51, 1.75)	0.8687267	0.8889296
denovo880054	Proteobacteria; Betaproteobacteria; Burkholderiales; Alcaligenaceae; Sutterella	Genus	430.87	6.92	2.48 (1.79, 3.42)	3.57E-08	9.39E-07	0.88 (0.47, 1.66)	0.6909691	0.7971924
denovo840450	Proteobacteria; Gammaproteobacteria; Pasteurellales	Order	14.4	1.08	0.73 (0.45, 1.18)	0.1971867	0.5176152	3.85 (1.53, 9.7)	0.0042946	0.0450935
denovo840450	Proteobacteria; Gammaproteobacteria; Pasteurellales; Pasteurellaceae	Family	13.38	0.19	0.69 (0.43, 1.11)	0.1221742	0.3839762	4.26 (1.72, 10.51)	0.0016864	0.0185508
denovo840450	Proteobacteria; Gammaproteobacteria; Pasteurellales; Pasteurellaceae; Haemophilus	Genus	13.31	0.17	0.77 (0.47, 1.25)	0.2885131	0.6331259	3.69 (1.48, 9.17)	0.0049722	0.0491005
denovo789089	Tenericutes; Mollicutes; RF39	Order	40.35	0.03	0.45 (0.23, 0.9)	0.0227849	0.1594944	0.19 (0.06, 0.67)	0.0095592	0.0501856
denovo789089	Tenericutes; Mollicutes; RF39; NA	Family	35.68	0.04	0.47 (0.24, 0.92)	0.0266385	0.1939091	0.21 (0.06, 0.71)	0.0118074	0.0649406
denovo789089	Tenericutes; Mollicutes; RF39; NA; NA	Genus	36.48	0.05	0.45 (0.23, 0.89)	0.021111	0.1667766	0.23 (0.07, 0.76)	0.0161438	0.0971602

Supplementary Table 8. Sensitivity analysis - excluding participants (n=5) who collected their stool sample <2 weeks after their colonoscopy.

	CA cases (n=144) vs. controls (n=319)		HP cases (n=39) vs. controls (n=319)		SSA cases (n=33) vs. controls (n=319)	
**α-diversity**[a]	**B (95% CI)**[b]	**P-value**[b]	**B (95% CI)**[b]	**P-value**[b]	**B (95% CI)**[b]	**P-value**[b]
Richness	-31.0 (-56.1, -5.9)	0.02	30.6 (-11.2, 72.5)	0.15	-3.1 (-47.8, 41.5)	0.89
Shannon index	-0.13 (-0.26, 0.00)	0.06	0.17 (-0.04, 0.39)	0.12	0.01 (-0.23, 0.24)	0.96
**β-diversity**		**P-value**[c]		**P-value**[c]		**P-value**[c]
Unweighted UniFrac		0.06		0.33		0.73
Weighted UniFrac		0.16		0.73		0.82
JSD		0.08		0.73		0.86
**Selected taxon abundance**	**Fold change (95% CI)**[d]	**P-value**[d]	**Fold change (95% CI)**[d]	**P-value**[d]	**Fold change (95% CI)**[d]	**P-value**[d]
Bacilli	2.07 (1.58, 2.72)	1.30E-07	1.25 (0.82, 1.9)	0.30	0.84 (0.54, 1.31)	0.44
Erysipelotrichi	0.91 (0.79, 1.06)	0.24	1.05 (0.82, 1.35)	0.68	0.69 (0.53, 0.89)	0.005
Gammaproteobacteria	1.78 (1.2, 2.63)	0.004	0.42 (0.24, 0.74)	0.003	0.51 (0.28, 0.92)	0.03
Enterobacteriales	2.06 (1.24, 3.43)	0.005	0.22 (0.1, 0.51)	0.0004	0.45 (0.19, 1.09)	0.08
Actinomyces	1.7 (1.33, 2.19)	2.86E-05	1.2 (0.79, 1.82)	0.39	1.07 (0.7, 1.64)	0.75
Streptococcus	2.38 (1.76, 3.22)	2.00E-08	1.28 (0.78, 2.09)	0.32	0.72 (0.43, 1.22)	0.22

[a]These indices were calculated for 500 iterations of rarefied (4000 sequences per sample) OTU tables, and the average over the iterations was taken for each participant (1 control excluded due to sequencing depth=2088).
[b]Betas and p-values for difference between cases and controls from multiple linear regression, adjusting for age, sex, categorical BMI, and study.
[c]P-values for difference between cases and controls from PERMANOVA of the distance matrix, adjusting for age, sex, categorical BMI, and study.
[d]Fold changes and p-values from DESeq2 models adjusted for age, sex, categorical BMI, and study.

Supplementary Table 9. Sensitivity analysis - excluding participants (n=19 from the NYU study) who had taken antibiotics within 30 days prior to sample collection (antibiotic usage information was not available in the CDC study).

	CA cases (n=135) vs. controls (n=314)		HP cases (n=39) vs. controls (n=314)		SSA cases (n=33) vs. controls (n=314)	
**α-diversity**[a]	**B (95% CI)**[b]	**P-value**[b]	**B (95% CI)**[b]	**P-value**[b]	**B (95% CI)**[b]	**P-value**[b]
Richness	-31.2 (-57.1, -5.3)	0.02	36.7 (-5.5, 78.9)	0.09	-1.6 (-46.7, 43.4)	0.94
Shannon index	-0.12 (-0.26, 0.01)	0.08	0.21 (-0.01, 0.43)	0.07	0.02 (-0.22, 0.25)	0.89
**β-diversity**		**P-value**[c]		**P-value**[c]		**P-value**[c]
Unweighted UniFrac		0.12		0.31		0.75
Weighted UniFrac		0.15		0.61		0.80
JSD		0.12		0.76		0.85
**Selected taxon abundance**	**Fold change (95% CI)**[d]	**P-value**[d]	**Fold change (95% CI)**[d]	**P-value**[d]	**Fold change (95% CI)**[d]	**P-value**[d]
Bacilli	2.09 (1.57, 2.77)	3.10E-07	0.9 (0.57, 1.42)	0.65	0.79 (0.48, 1.28)	0.33
Erysipelotrichi	0.92 (0.79, 1.08)	0.32	1.09 (0.85, 1.4)	0.48	0.68 (0.52, 0.89)	0.004
Gammaproteobacteria	1.69 (1.1, 2.59)	0.02	0.31 (0.16, 0.62)	0.0008	0.41 (0.2, 0.85)	0.02
Enterobacteriales	1.9 (1.13, 3.19)	0.02	0.21 (0.09, 0.49)	0.0002	0.43 (0.18, 1.03)	0.06
Actinomyces	1.7 (1.32, 2.18)	4.04E-05	1.08 (0.71, 1.64)	0.73	1.06 (0.69, 1.63)	0.78
Streptococcus	2.43 (1.79, 3.3)	1.00E-08	0.85 (0.52, 1.38)	0.51	0.72 (0.43, 1.21)	0.22

[a]These indices were calculated for 500 iterations of rarefied (4000 sequences per sample) OTU tables, and the average over the iterations was taken for each participant (1 control excluded due to sequencing depth=2088).
[b]Betas and p-values for difference between cases and controls from multiple linear regression, adjusting for age, sex, categorical BMI, and study.
[c]P-values for difference between cases and controls from PERMANOVA of the distance matrix, adjusting for age, sex, categorical BMI, and study.
[d]Fold changes and p-values from DESeq2 models adjusted for age, sex, categorical BMI, and study.
